# TRIM32 modulates pluripotency entry and exit by directly regulating Oct4 stability

**DOI:** 10.1038/srep13456

**Published:** 2015-08-26

**Authors:** Lamia’a Bahnassawy, Thanneer M. Perumal, Laura Gonzalez-Cano, Anna-Lena Hillje, Leila Taher, Wojciech Makalowski, Yutaka Suzuki, Georg Fuellen, Antonio del Sol, Jens Christian Schwamborn

**Affiliations:** 1 Westfälische Wilhelms-Universität Münster, ZMBE, Institute of Cell Biology, Stem Cell Biology and Regeneration Group, Von-Esmarch-Str. 56, 48149 Münster, Germany; 2 Luxembourg Centre for Systems Biomedicine (LCSB), Developmental and Cellular Biology, University of Luxembourg, 7 avenue des Hauts-Fourneaux, 4362 Esch-Belval, Luxembourg; 3 Luxembourg Centre for Systems Biomedicine (LCSB), Computational Biology, University of Luxembourg, 7 avenue des Hauts-Fourneaux, 4362 Esch-Belval, Luxembourg; 4 Institute for Biostatistics and Informatics in Medicine und Ageing Research, Rostock University Medical Centre, Ernst-Heydemann-Str. 8, 18057 Rostock, Germany; 5 Westfälische Wilhelms-Universität Münster, Institute of Bioinformatics, Niels-Stensen-Straße 14, 48149 Münster, Germany; 6 Department of Medical Genome Sciences, University of Tokyo, 5-1-5 Kashiwanoha, Kashiwa-shi, Chiba-ken 227-8561, Japan

## Abstract

Induced pluripotent stem cells (iPSCs) have revolutionized the world of regenerative medicine; nevertheless, the exact molecular mechanisms underlying their generation and differentiation remain elusive. Here, we investigated the role of the cell fate determinant TRIM32 in modulating such processes. TRIM32 is essential for the induction of neuronal differentiation of neural stem cells by poly-ubiquitinating cMyc to target it for degradation resulting in inhibition of cell proliferation. To elucidate the role of TRIM32 in regulating somatic cell reprogramming we analysed the capacity of TRIM32-knock-out mouse embryonic fibroblasts (MEFs) in generating iPSC colonies. TRIM32 knock-out MEFs produced a higher number of iPSC colonies indicating a role for TRIM32 in inhibiting this cellular transition. Further characterization of the generated iPSCs indicated that the TRIM32 knock-out iPSCs show perturbed differentiation kinetics. Additionally, mathematical modelling of global gene expression data revealed that during differentiation an Oct4 centred network in the wild-type cells is replaced by an E2F1 centred network in the TRIM32 deficient cells. We show here that this might be caused by a TRIM32-dependent downregulation of Oct4. In summary, the data presented here reveal that TRIM32 directly regulates at least two of the four Yamanaka Factors (cMyc and Oct4), to modulate cell fate transitions.

Mouse embryonic fibroblasts (MEFs) have been successfully reprogrammed into induced pluripotent stem cells (iPSCs) through the ectopic expression of the transcription factors Oct4, Sox2, Klf4, and cMyc (OSKM)^1^. Exploring this potential further, several groups achieved similar reprogramming from various cell types and species such as human fibroblasts^1^,^2^, human adipose tissue^3^ and human peripheral blood cells^4^, to mention some^5^. Furthermore, various transgene delivery methods evolved. The use of episomal vectors^6^ and the direct delivery of mRNA^7^ or protein^8^ of the four factors are some examples. Exploring the molecular mechanisms underlying the reprogramming process in more detail revealed a wide variety of factors that can substitute for OSKM^9^,^10^,^11^, such as other family members of the individual transcription factors^12^, or expression of certain microRNA (miRNA) clusters^13^,^14^. Nevertheless, the efficiency of generation of iPSCs varies between the different methods and the different factors used in the process^5^,^15^. Furthermore, a series of cellular modulators have been outlined to either improve or block the reprogramming process^16^,^17^,^18^,^19^,^20^,^21^. This is indicative of the necessity for a deeper understanding of how iPSCs arise and how these cells differentiate into cells of the different germ layers^22^,^23^.

TRIM32 is a ubiquitously expressed E3 ubiquitin ligase with highest expression levels in the brain^24^,^25^ and belongs to the tripartite motif (TRIM-NHL) family of proteins^26^. The E3 ubiquitin ligase function resides in its RING-domain at the N-terminus^27^,^28^,^29^. Additionally the NHL-domain at the C-terminus mediates the interaction with Argonaute proteins and consequently allows TRIM32 to modulate the activity of certain miRNAs^30^. TRIM32 has been shown to be important for neuronal differentiation of neural progenitor cells^30^,^31^,^32^. The underlying molecular mechanism involves both functionalities of TRIM32. On the one hand, TRIM32 ubiquitinates cMyc -an essential factor for the proliferation of neural stem cells^32^,^33^,^34^ -and targets it thereby for proteasomal degradation. On the other hand, TRIM32 enhances the activity of the miRNA Let-7a, through its interaction with Argonaute proteins. This property of TRIM32 that allows it to control cell fate is not only confined to the nervous system but also extends to skeletal muscle progenitor cells^35^.

Despite the described role for TRIM32 in the development of the nervous system and the muscle, it remains unknown whether TRIM32 also plays a role earlier in development. It is particularly tempting to speculate that TRIM32 is also able to modulate the function of pluripotent stem cells. A role in pluripotency regulation as well as modulation of cellular reprogramming has been shown for other TRIM-NHL family members^36^,^37^. Considering the so far described functions of TRIM32 it is expected that it constitutes a hurdle for cellular reprogramming into pluripotency and that consequently its absence would facilitate iPSCs generation. Therefore, we aimed at investigating the expression pattern of TRIM32 in pluripotent cells as well as in differentiated cells. In addition, we assayed the reprogramming capacities of TRIM32 knock-out (TRIM32-ko) MEFs and investigated the potential of TRIM32 to regulate the gene-regulatory networks governing pluripotency and differentiation.

The data presented here show that the absence of TRIM32 improves the reprogramming efficiency of MEFs into iPSCs. Moreover careful analysis of global gene expression of TRIM32-ko iPSCs and thereof derived differentiated cells shows that the route that is taken by these cells to achieve differentiation differs significantly from their wild-type counterparts. At the core of these differences is the differential role of Oct4. Interestingly we show that TRIM32 can regulate the stability of Oct4. In conclusion, our data support the fact that TRIM32 is a pluripotency-reprogramming roadblock that facilitates cellular transition towards differentiation via modulating the levels of Oct4 and cMyc.

## Results

### TRIM32 is expressed in pluripotent stem cells

**Figure 1:**
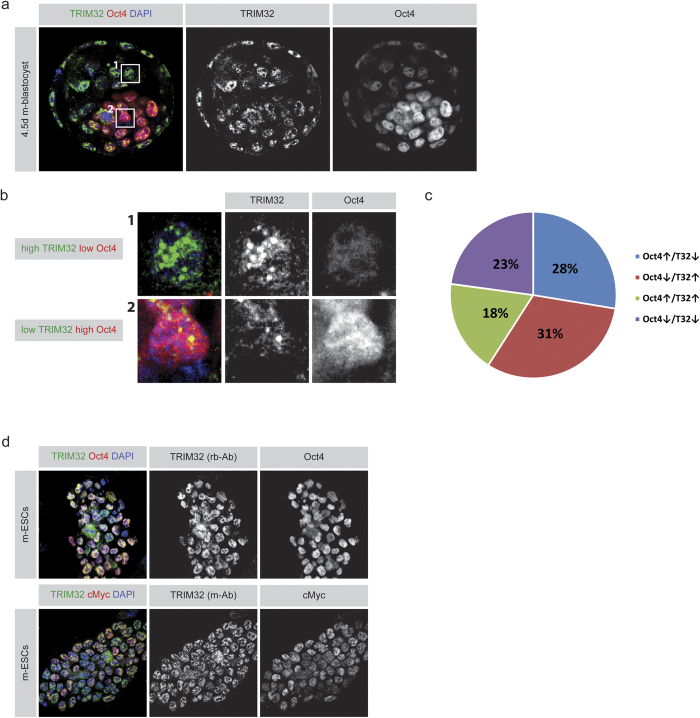
TRIM32 is expressed in pluripotent stem cells (**a**) Immunostainings of 4.5 days old mouse blastocysts labelled for TRIM32 (abbreviated T32) and Oct4. (**b**) Magnification of individual cells from panel (**a). (c**) Quantification of the immunofluorescence intensities of TRIM32 and Oct4 in individual cells of the blastocyst. Cells from three different z-layers per blastocyst were measured and in total at least three blastocysts were quantified. All cells were measured except for the trophoectoderm cells. Cells were divided into the four indicated groups. Cells with intensities above the overall average intensity were considered as “high” and cells below were considered as “low”. (**d**) Immunostainings of C57BL/6 mESCs stained for the indicated markers (upper boxes).

The expression pattern of TRIM32 in pluripotent stem cells has not yet been investigated in detail. Hence, we first analysed the protein expression pattern of TRIM32 in E4.5 old wild-type mouse blastocysts. We used specific antibodies to detect TRIM32 and the transcription factor Oct4 which marks cells of the inner cell mass (ICM)^38^. We found TRIM32 to be expressed in blastocysts (Fig. 1a). Its expression is not confined to a specific cellular population in contrast to Oct4, which is mainly expressed in the ICM. Nevertheless, the expression level of TRIM32 varies between individual cells of the blastocyst; some cells showed high TRIM32 and low Oct4 levels while others have low TRIM32 and high Oct4 levels (Fig. 1a,b). This expression pattern suggests a possible counter regulation of both proteins. However, since cells with simultaneously high or low levels of both proteins were detectable, the transitions between these states might be rather dynamic (Fig. 1c).

Further, the expression of TRIM32 in pluripotent stem cells was ascertained by staining for TRIM32, along with a panel of pluripotency markers, in cultured mouse embryonic stem cells (mESCs). It was found that these cells expressed TRIM32 in the nucleus, a pattern that we confirmed using two different antibodies (Fig. 1d). The cells additionally express Oct4, cMyc, Sox2, Nanog and Lin28 (Fig. 1d and Suppl Figure 1a), which are markers of mESCs^1^,^39^. Notably in these cells, TRIM32 is more uniformly expressed with no obvious level variations as in the blastocyst. The data supports the expression of TRIM32 in pluripotent stem cells where it is confined to the nuclear compartment.

### TRIM32 expression levels increase with cellular differentiation

**Figure 2:**
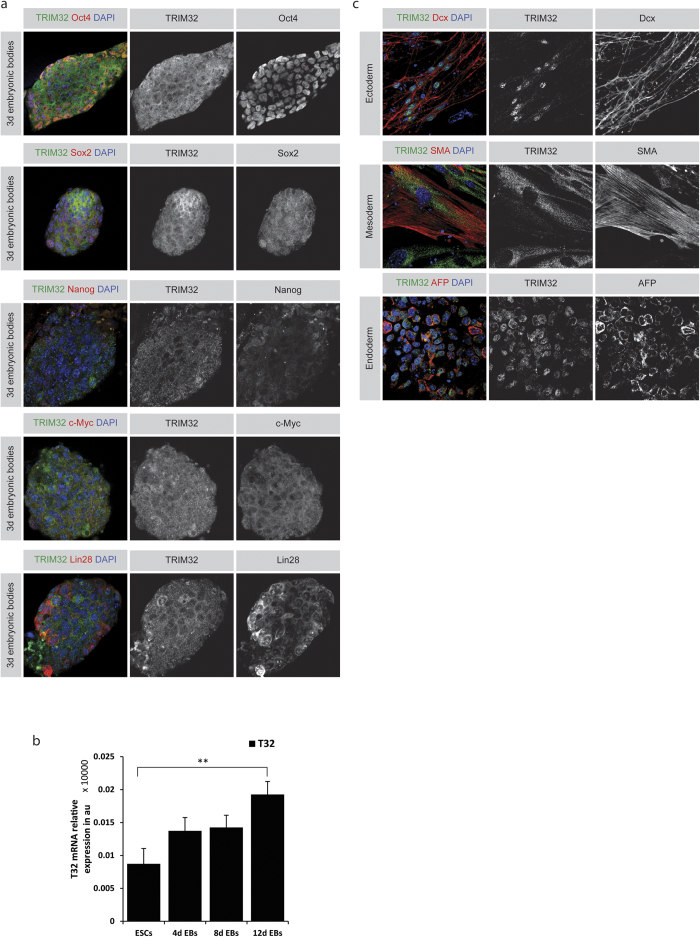
TRIM32 expression increases in differentiating cells (**a**) Immunostainings of 3 day old embryonic bodies (EBs) for the indicated markers (upper boxes). EBs were generated from C57BL/6 mESCs and grown in ESC media without LIF for 3 days. (**b**) RT-qPCR for TRIM32 mRNA levels in differentiating EBs. EBs were generated as described above and allowed to grow for 12 days. RNA was isolated every 4 days. Represented are averages from at least three independent experiments in arbitrary units (au). Error bars represent SEM. Statistical analysis was performed using t-test or a Mann-Whitney rank sum test (*p ≤ 0.05, **p ≤ 0.01). (**c**) Immunostainings of 14 day long differentiated C57BL/6 mESCs. EBs were generated from C57BL/6 mESCs and grown in ESC media without LIF for 3 days, after which they were seeded on Gelatine and allowed to grow till day 14. Cells were stained for the indicated markers (upper boxes).

Previous data have indicated an upregulation of the protein levels of TRIM32 in differentiating neural progenitor cells of the adult mouse brain. Furthermore, the differentiation process in these cells is also associated with changes in the subcellular distribution of TRIM32^31^. Consequently, we analysed the expression and localization of TRIM32 in mESCs that were grown under conditions that slowly induced their differentiation. For this purpose, mESCs were grown as embryonic bodies (EBs) in the absence of leukemia inhibitory factor (LIF) and RNA was isolated or the cells were immunofluorescently stained at the desired time points. Here, we have chosen particularly mild differentiation inducing conditions to be able to investigate the early stages of cell fate transitions. After three days of differentiation, most pluripotency markers were downregulated (Fig. 2a). Interestingly, TRIM32 was strongly expressed in these cells, but in contrast to the situation in mESCs under maintenance conditions, it was additionally localized in the cytoplasm of these cells (Fig. 2a). This could be an indicator for the probable acquisition of a new role by TRIM32 after initiation of differentiation for which it is shuttled between the different cellular compartments.

Furthermore, we quantified the mRNA levels of *TRIM32* via RT-qPCR at several time points during the differentiation process of mESCs into EBs. *TRIM32’s* expression increased two-fold after twelve days indicating that TRIM32 is gaining a more dominant function during differentiation. Although one would expect to see a stronger increase in the mRNA levels of *TRIM32*, this could be explained by the mild differentiation conditions used, which is also reflected on the mRNA levels of the pluripotency factors, such as *Oct4* and *Sox2* (Suppl Figure 1b–f). Of note is that although the protein expression of these markers was already clearly downregulated after three days (Fig. 2a), the mRNA seems to persist longer^40^.

Lastly, we analysed the expression of TRIM32 in cells of the three germ layers that are derived from differentiating mESCs. Ectodermal cells labelled with the young neuronal marker doublecortin x (Dcx) expressed TRIM32 in the nucleus as has been previously reported for neurons (Fig. 2c)^30^,^31^,^32^. Mesodermal cells that are smooth muscle actin (SMA) positive and endodermal cells expressing alpha-feto-protein (AFP) also express TRIM32. In conclusion, TRIM32 is expressed in pluripotent stem cells as well as in differentiated cells of all germ layers while having higher expression levels and a more dynamic subcellular distribution in differentiated cells.

### TRIM32 is a reprogramming roadblock for the generation of iPSCs

The fact that the expression levels of TRIM32 in differentiated EBs are higher than in pluripotent stem cells invites the hypothesis that the absence of TRIM32 would facilitate the generation of iPSCs. Indeed wild-type MEFs express significant levels of TRIM32 (Suppl Figure 2a). To further explore whether TRIM32 might act as a roadblock for reprogramming, we reprogrammed TRIM32 deficient MEFs into iPSCs using the classical Yamanaka factors approach and assessed the speed and efficiency of iPSC colony generation. For this purpose, four independent pairs of TRIM32-wild-type (TRIM32-wt) and TRIM32-ko MEFs were generated from four different embryo litters.

**Figure 3:**
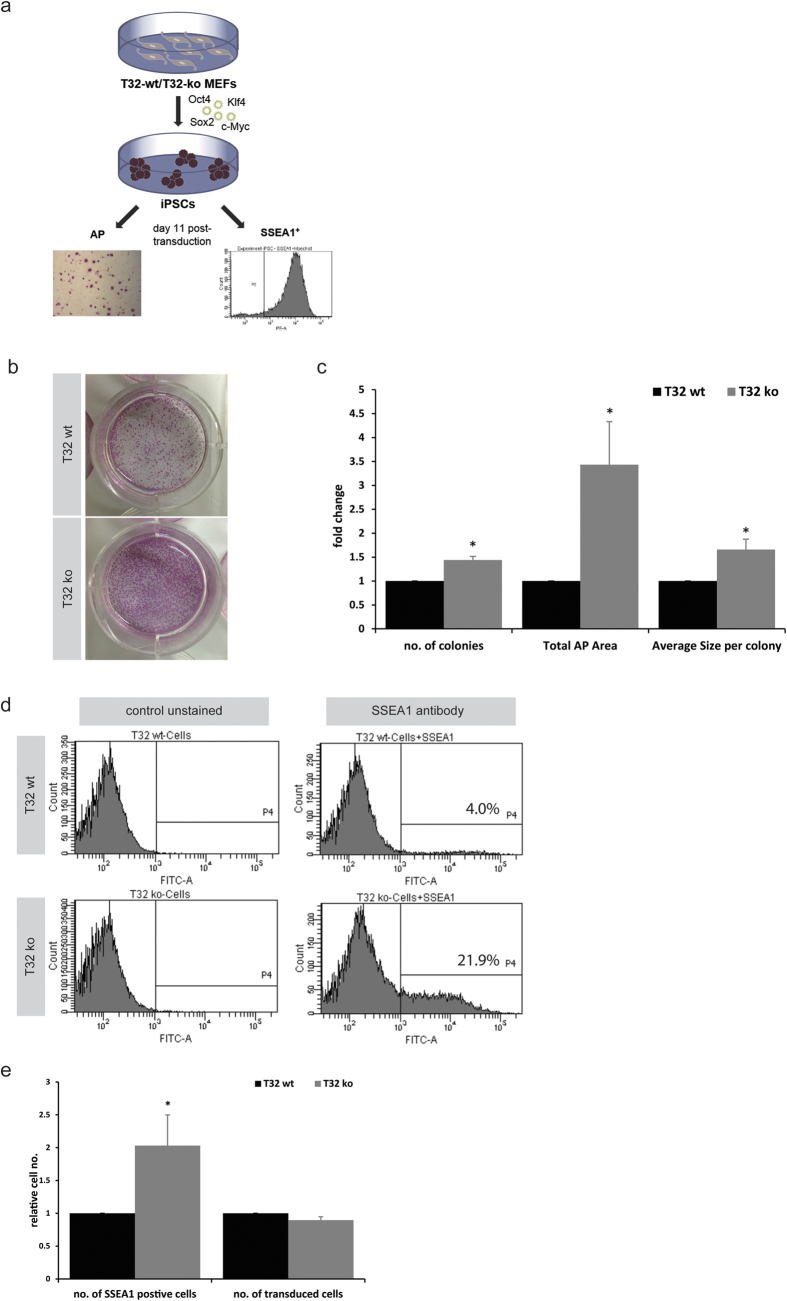
TRIM32 impedes somatic cell reprogramming (**a**) Experimental concept for the reprogramming of TRIM32-wt and TRIM32-ko MEFs. (**b**) Alkaline phosphatase staining of reprogrammed wild-type and TRIM32-ko MEFs at day 11 after viral transduction. (**c**) Quantification of the alkaline phosphatase staining. Quantification was performed using Image J to quantify number and size of the colonies as well as the total area occupied by alkaline phosphatase. Values represent averages from three individual experiments, during each four independent pairs of wt and ko MEFs were utilized. Values were normalized to the wt cells. Error bars represent SEM. Statistical analysis was performed using t-test (*p ≤ 0.05). (**d**) Flow cytometric analysis of SSEA1 positive cells. One million cells were stained per condition. Unstained cells were used as a negative control. (**e**) Quantification of the flow cytometric data. Values represent averages from 3 individual experiments, during each four independent pairs of wt and ko MEFs were utilized. Values were normalized to the wt cells. Error bars represent SEM. Statistical analysis was performed using Mann-Whitney rank sum test (*p ≤ 0.05).

The lines isolated from sibling embryos were compared and then averages were calculated among the four pairs of MEFs. For the reprogramming approach a lentiviral vector carrying all four factors was employed^41^ and efficiency was analyzed using alkaline phosphatase (AP) staining and flow cytometric analysis for the surface mouse stem cell marker SSEA1^42^ at day eleven after viral transduction (Fig. 3a).

As expected, TRIM32-ko MEFs generated more (approximately 50% more) AP positive colonies (Fig. 3b,c). Careful inspection of the colonies implicated that the TRIM32-ko MEFs had the tendency not only to produce more, but also larger colonies in comparison to the TRIM32-wt cells. Hence, we quantified the total AP occupied area, and the average size per colony, which confirmed that TRIM32 deficiency leads to the generation of larger iPSC colonies and therefore the AP occupied area was increased by around 3.5-folds (Fig. 3c).

This difference in size motivated us to question whether the difference is mediated by a higher number of partially reprogrammed cells or by true iPSCs. To address this question we stained the cells for SSEA1 and quantified the number of positively labelled cells by flow cytometry. Again, the TRIM32-ko MEFs produced on average a 2-fold higher amount of SSEA1 positive cells in comparison to their wild-type counterparts (Fig. 3d,e). In order to exclude the possibility that this is a result of differential viral transduction efficiencies we assessed the number of transduced cells, which is reflected by the expression of dTomato that is encoded by the viral plasmid^41^. Concerning the transduction efficiency, we did not observe any significant differences between the two groups (Fig. 3e) confirming that the observed increase in reprogramming efficiency is due to the absence of TRIM32. In addition, the expression of SSEA1 as well as Nanog by the reprogrammed cells at day eleven was confirmed via Immunofluorescence staining (Suppl Figure 2b) and Western Blotting of the generated iPSCs against Oct4, Sox2 and Nanog (Suppl Figure 2c). In summary, the absence of TRIM32 facilitates the generation of iPSCs from MEFs indicating that TRIM32 acts as a cellular reprogramming barrier.

### TRIM32 is not required for the maintenance of the pluripotent state

**Figure 4:**
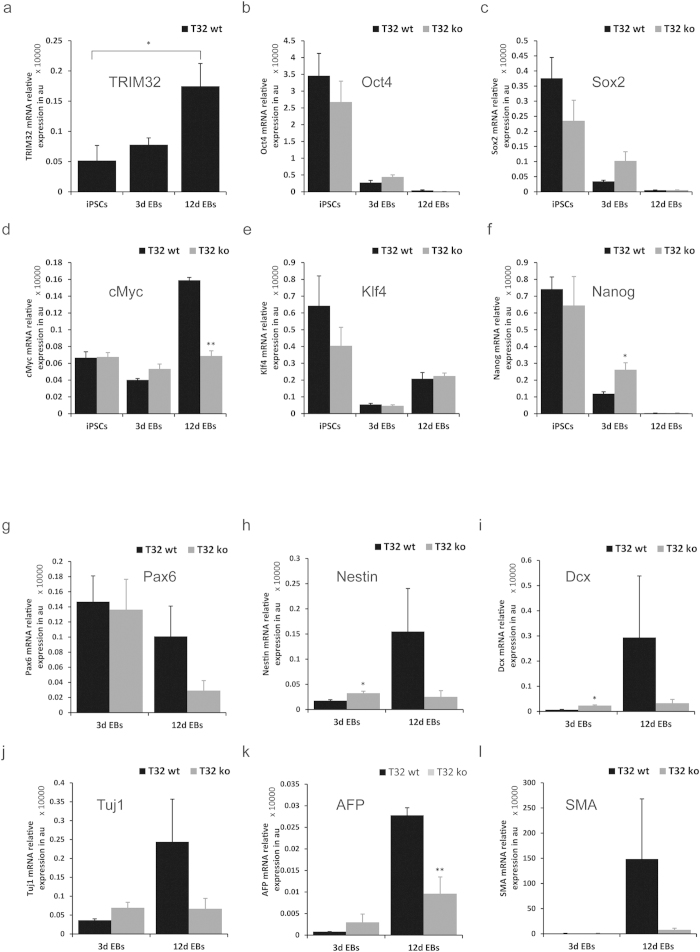
TRIM32-ko iPSC mRNA expression patterns. RT-qPCR data from iPSCs, 3 day old EBs and 12 day old EBs representing relative expression of the indicated genes. Values were normalized to GAPDH levels and are expressed in arbitrary units. Displayed are averages ± SEM and statistical analysis was performed using either a t-test or a Mann-Whitney rank sum test (*p ≤ 0.05, **p ≤ 0.01).

To follow up on analysing the role of TRIM32 in pluripotent stem cells, iPSC clones were picked from both the TRIM32-wt and TRIM32-ko reprogrammed MEFs. Two independent pairs of clones were used for further downstream analysis (Suppl Figure 3a). Silencing of the exogenously introduced genes was confirmed via RT-qPCR in all clones (Suppl Figure 3b). Morphologically, the TRIM32-ko iPSCs did not show any obvious differences compared to their wt counterparts (Suppl Figure 3a). Immunofluorescence staining for pluripotency markers in both cell lines confirmed similar levels of expression of the major pluripotency markers such as Oct4, Nanog and Lin28 as well as the cell cycle marker Ki67 (Suppl Figure 3c). Quantifying for mRNA levels of the same markers via RT-qPCR confirmed the absence of any difference with regards to these markers between the TRIM32-wt and TRIM32-ko cells under maintenance conditions (Fig. 4b–f).

To examine whether these cells are true iPSCs, they were allowed to differentiate into EBs in absence of LIF. Both TRIM32-wt and TRIM32-ko iPSCs were able to generate EBs (Suppl Figure 3d) that had the potential to produce cells of all germ layers as indicated by the presence of Dcx, SMA and AFP positive cells (Suppl Figure 3e).

These data demonstrate that TRIM32-ko MEFs are capable of generating iPSCs that have the potential to differentiate into all germ layers. Additionally, TRIM32 does not seem to play an obvious role in the maintenance of these iPSCs in a pluripotent state.

### TRIM32-ko iPSCs display altered differentiation behaviour and kinetics

Despite the fact that TRIM32-ko iPSCs could generate cells of all germ layers, it is interesting to quantitatively assess their differentiation capabilities. Because of the described function of TRIM32 in neuronal differentiation of neural stem cells^30^,^31^, it was of special interest to investigate the speed and efficiency by which TRIM32 deficient iPSCs achieve commitment into the ectodermal lineage. This was analysed using TRIM32-wt and TRIM32-ko iPSCs grown as EBs in absence of LIF and with the addition of retinoic acid to induce neuro-ectodermal differentiation (Suppl Figure 4a). Loss of pluripotency as well as differentiation was assessed after three and twelve days. mRNA was isolated at these two time points and subjected to RNA-sequencing and was also used for RT-qPCR analysis of specific markers. Moreover, cells were plated for Immunofluorescence staining also after three and twelve days of differentiation to elucidate the expression of pluripotency markers as well as differentiation markers.

After twelve days of differentiation the mRNA levels of *TRIM32* in the TRIM32-wt iPSCs displayed a significant increase (Fig. 4a). TRIM32-ko iPSCs showed a consistent tendency for having higher mRNA levels of the pluripotency factors especially at day three of differentiation (Fig. 4b–f). This difference was most prominent in the case of *Sox2* and *Nanog* (Fig. 4c,f). Nevertheless, these differences seem to level out after twelve days of differentiation, where cells of both genotypes downregulate *Oct4*, *Sox2* and *Nanog* (Fig. 4b,c,f). Interestingly, the mRNA levels of *cMyc* of the TRIM32-wt iPSCs show a striking increase after twelve days whereas the TRIM32-ko cells keep a steady state throughout differentiation (Fig. 4d). This could be a reflection of TRIM32 mediated decrease in cMyc protein levels in the wt cells, since cMyc protein has been shown to negatively regulate its own mRNA levels in a feedback mechanism^43^. The expression level of *Klf4* is quite similar in both lines showing an initial decrease after three days and a slight increase after twelve days (Fig. 4e).

Within this context, the expression of differentiation markers was analysed with more emphasis on those involved in neuro-ectodermal lineage determination. Most of the prominent differences became apparent only after twelve days of differentiation (Fig. 4g–l). With the exception of *Pax6* (Fig. 4g), the TRIM32-ko cells seem to have persistently low mRNA levels of these markers that fail to increase after twelve days of differentiation. In contrast, the TRIM32-wt cells upregulate the mRNA for *Nestin* (Fig. 4h), *Dcx* (Fig. 4i) and *Tuj1* (Fig. 4j). Similar effects are detectable for the endoderm and mesoderm markers *AFP* (Fig. 4k) and *SMA* (Fig. 4l). Yet, the variability in the TRIM32-wt cells is extremely high, so that most of these differences are not statistically significant in comparison to the TRIM32-ko cells. Remarkably, this high variability was not observed in the case of the TRIM32-ko cells, denoting that these cells have a constant lower ability to differentiate. In conclusion, the TRIM32-ko cells show impaired differentiation as shown by the slower downregulation of the pluripotency markers and the failure to upregulate differentiation markers (Fig. 4).

**Figure 5:**
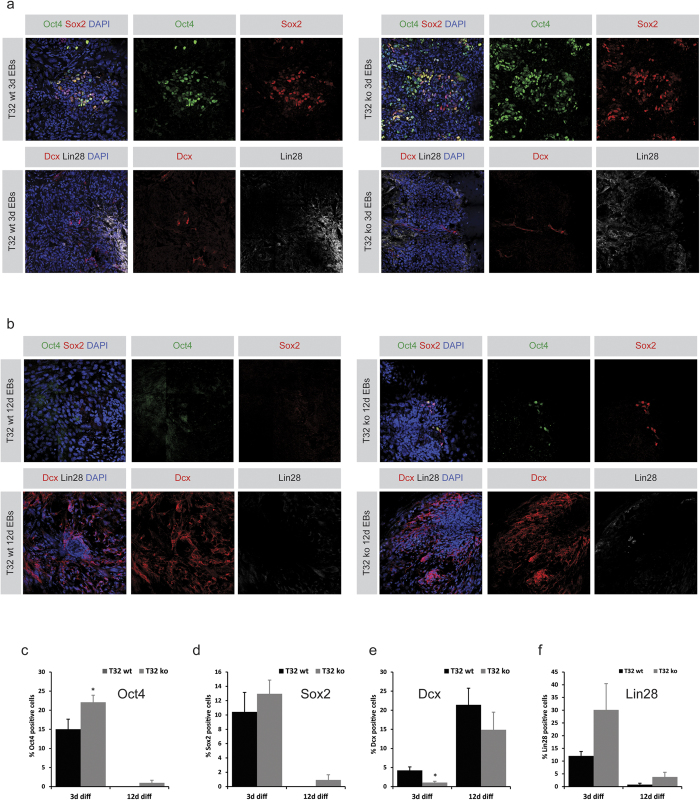
TRIM32-ko iPSCs the abbreviation was introduced before display perturbed differentiation kinetics (**a**) Immunostainings of 3 day old EBs for the indicated markers (upper boxes). EBs were generated from either TRIM32-wt iPSCs (left) or TRIM32-ko iPSCs (right) and were grown in ESC media without LIF with 5 μM retinoic acid for 3 days after which they were allowed to adhere to Matrigel coated cover slips overnight. Images show 3 × 3 tile-scans. (**b**) Immunostainings of 12 day old EBs for the indicated markers (upper boxes). EBs were generated from either TRIM32-wt iPSCs (left) or TRIM32-ko iPSCs (right) and were grown in ESC media without LIF with 5 μM retinoic acid for 12 days. They were seeded on Matrigel coated coverslips on day 6. Images show 3 × 3 tile-scans. (**c–f)** Quantification of the percentage of positive cells for the indicated markers in (**a,b)** after 3 and 12 days of differentiation. Values represent averages from two independent clones for both TRIM32-wt and TRIM32-ko cells. At least 3 3 × 3 tile-scans were quantified per condition i.e. no. of cells counted ≥200 per cell line and per condition. Statistical analysis was performed based on the no. of tile-scans quantified using a t-test or a Mann-Whitney rank sum test (*p ≤ 0.05). Displayed are averages in percentage ± SEM.

Since TRIM32 is an E3-ubiquitin ligase^27^, it might also exert effects on protein ubiquitination and degradation, and consequently further differences between the two cell lines might be revealed when looking at immunofluorescent labelling of the various markers throughout differentiation. A quantification of the amount of Oct4 positive cells revealed that the TRIM32-ko cells had around 10% more Oct4-positive cells after three days of differentiation in comparison to the TRIM32-wt cells (Fig. 5a,c). Interestingly, even after twelve days the TRIM32-ko cells showed residual Oct4 expression whereas it had completely disappeared from the TRIM32-wt cells (Fig. 5b,c). A similar tendency was observed for Sox2 (Fig. 5a,b,d), Lin28 (Fig. 5a,b,f) and Nanog (Suppl Figure 4b,c and d), yet these differences were not statistically significant. Nevertheless, these results support the tendencies observed at the mRNA level. Although we did not observe any differences for the mRNA levels of *Klf4*, the percentage of Klf4 positive cells is increased in the TRIM32-ko cells after twelve days of differentiation (Suppl Figure 4b, c and g). It has been previously shown that downregulation of Klf4 is essential for proper embryonic neurogenesis^44^. Hence, the higher levels of Klf4 in the TRIM32-ko cells could contribute to the decreased neuronal maturation of these cells.

Quantifying for early neuronal markers, no difference was detected in the number of Sox1 positive cells (Suppl Figure 4b,c and f) while the amount of Pax6 positive cells reflected the pattern seen on the mRNA level (Suppl Figure 4b,c and h). The amount of Dcx positive cells after three days of differentiation was significantly lower in the TRIM32-ko cells (Fig. 5a,e). Although the ko cells retain this tendency after twelve days, they seem to be able to catch up with the TRIM32-wt cells (Fig. 5b,e). The tendencies for Tuj1 after twelve days were similar. (Suppl Figure 4b,c and i). Altogether, these results support a concept where TRIM32 acts as a modulator of cell fate transitions, rather than as a determinant of a certain cell fate or stage.

Concerning the amount of SMA (Suppl Figure 4b,c and e) and AFP positive cells (Suppl Figure 4b,c and j) no obvious differences could be observed despite the detected mRNA tendencies. This supports the more dominant role of TRIM32 in regulating ectodermal lineage commitment.

### Functional annotation of differentially expressed genes suggests neuronal differentiation and neuron fate commitment to be differentially regulated in the TRIM32-ko cells

To obtain a more global perspective on the effect of the loss of TRIM32 on iPSCs maintenance and differentiation, total mRNA from iPSCs, 3 days and 12 days differentiated EBs was subjected to high-throughput RNA-seq analysis. Clustering of global gene expression patterns showed that the stages of differentiation agreeably cluster together (Suppl Figure 5a). However, the euclidian distance between wild-type and knock-out expression profiles increases from iPSCs to 3 days to 12 days old EBs suggesting an amplified effect of the absence of TRIM32 as differentiation progresses. Accordingly, a higher number of genes were differentially expressed after 12 days of differentiation in comparison to 3 days of differentiation and iPSCs (Suppl Figure 5b). A total of 2250, 2173 and 5444 genes out of 16617 genes were differentially expressed between TRIM32-wt and TRIM32-ko cell lines in iPSCs, 3 days EBs and 12 days EBs, at a 5% false discovery rate, respectively.

Differentially expressed genes in the above analysis were interpreted using a functional annotation analysis for GO-terms and KEGG pathways using DAVID Bioinformatics software^45^. Comparison of the TRIM32-wt and TRIM32-ko iPSCs showed lack of any significant representation for major pathways. Interestingly, analysis at 3 days EBs gave a different picture. It demonstrated enrichment for biological processes such as neuronal differentiation, neuron fate commitment, and neuron fate determination along with many transcriptional regulation processes. These processes disappear after 12 days of differentiation and a small number of over represented genes for female pregnancy and stem cell differentiation processes appear.

### Mathematical modelling of gene expression analysis identifies different core regulatory networks responsible for the differentiation of TRIM32-wt and TRIM32-ko iPSCs

In order to elucidate the key regulatory mechanisms of cellular transitions, from iPSCs to 3 days EBs to 12 days EBs, gene regulatory networks (GRNs) followed by Boolean modelling were inferred from the top 2000 differentially expressed genes and 500 differentially expressed transcription factors. GRNs and Boolean models were generated for four different conditions, namely transition from iPSCs to 3 days EBs in the TRIM32-wt and TRIM32-ko cells and similarly for the transition from iPSCs to 12 days EBs of both genotypes.

**Figure 6:**
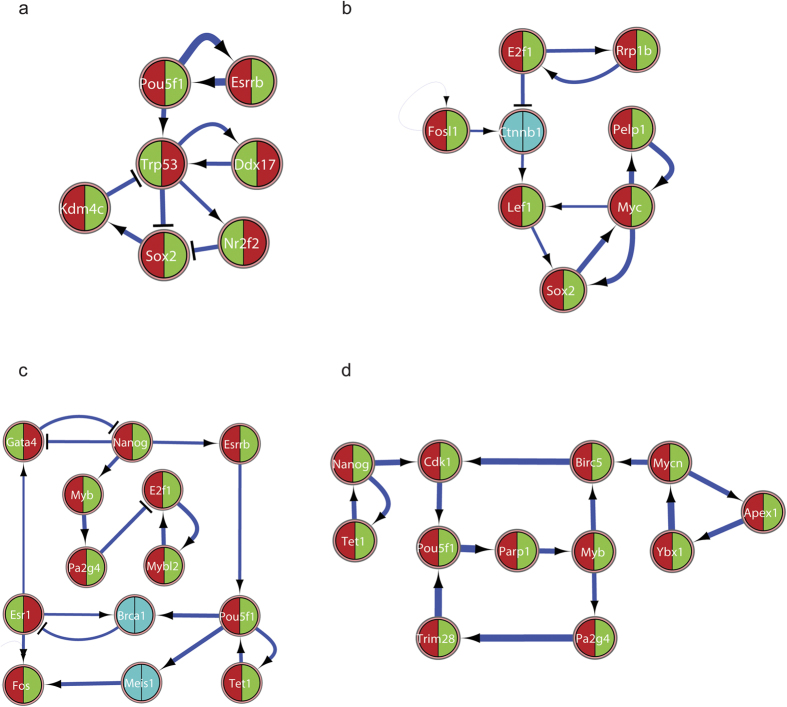
The cores of GRNs are controlled differently for TRIM32-wt and TRIM32-ko iPSC differentiation to 3 days and 12 days EBs (**a**) *Core of GRN for TRIM32-wt iPSC differentiation to 3 day EBs.* Each node represents a gene/protein of interest and each interaction represents an activation or inhibition that are either inferred from literature or obtained from expression data. Each node is divided in two halves and colored. The left and right hand partitions represent expression patterns in iPSCs and 3 day EBs respectively, and the color codes green or red represent down or up regulation of expression. If the node is colored blue, then it is either not significantly expressed for a FDR of 5% or mis-inferred in our model, and hence not used in our analysis. These rules apply to all the following networks. (**b**) *Core of GRN for TRIM32-ko iPSC differentiation to 3 day EBs.* (**c**) *Core of GRN for TRIM32-wt iPSC differentiation to 12 day EBs.* (**d**) *Core of GRN for TRIM32-ko iPSC differentiation to 12 day EBs.*

Transition of TRIM32-wt iPSCs to 3 days EBs resulted in a GRN model with 190 genes and 296 interactions with close to 83% predictability of differential expression (Suppl Figure 5c). Subsequent differential stability analysis^46^ of the resulting core of the GRN model elucidated the following mechanisms of cellular transition: a differentially expressed mutually activating feedback loop of *Oct4* and *Esrrb* regulating an intertwined downstream positive feedback loop between *Sox2*, *Nr2f2*, and *p53* along with *Kdm4c* as the regulator of differentiation (Fig. 6a) suggesting a potential role of H3-K9 de-methylation by Kdm4c and Oct4-Sox2 based activation of differentiation processes. Similarly, the model for the TRIM32-ko condition resulted in 258 genes and 424 interactions with close to 82% predictability of differential expression (Suppl Figure 5d). In contrast to the differential stability analysis of wild-type cells, the core regulatory network in the knock-out cells suggests an *E2f1* mediated regulation of DNA damage checkpoint control and DNA replication as the triggering mechanism for differentiation. Specifically, this is achieved by a mutual activating feedback loop between *E2F1* and *Rrp1b*, which in turn inhibits the positive feedback loop of *cMyc*, S*ox2*, *Lef1* and *Pelp1* genes (Fig. 6b).

Analogous analysis of the transition of iPSCs to 12 days EBs of TRIM32-wt and TRIM32-ko cells resulted in a different GRN model. The wild-type cells lead to a Boolean model with 274 genes and 460 interactions with close to 84% predictability of differential expression (Suppl. Figure 5e), and the knock-out cells lead to a Boolean model with 289 genes and 317 interactions with close to 82% predictability of differential expression (Suppl Figure 5f). In agreement with our differential stability analysis at 3 days, a mutually activating loop, involving *Oct4–Tet1,* continues to regulate the cellular transition of wild-type cells until 12 days of differentiation (Fig. 6c). However, these genes are regulated by a mutually repressing feedback loop involving *Gata4* and *Nanog*. Likewise, the analysis of TRIM32-ko iPSCs transition to the 12 day time point is mediated by *E2F1* transcriptional regulation and DNA damage pathways similar to the 3 days stage. This is resulted by a coupled feedback mechanism between *Oct4*, *Parp1*, *Myb*, *Pa2g4*, *TRIM28* and *Oct4*, *Parp1*, *Myb*, *Birc5*, *Cdk1* (Fig. 6d), which in turn is regulated by *Nanog*-*Tet1* and *Mycn*, *Ybx1* and *Apex1*. These genes are known to regulate E2F1 mediated DNA repair during G2/M phase transition.

Overall, the model based analysis of the global gene expression data show that there are none or negligible differences in the transcriptome of TRIM32-wt and TRIM32-ko iPSCs. However, major changes are seen during the differentiation process. The Boolean modelling of this data suggests a regular *Oct4* mediated differentiation in the TRIM32-wt cell lines when compared to an *E2F1* regulation for differentiation in the TRIM32-ko cell lines.

### TRIM32 destabilizes Oct4

The modelling of the differentially expressed genes strongly suggests that the *Oct4* centred network, which regulates differentiation in wild-type conditions, is absent in the TRIM32-ko cells. Combining this observation with the effect on the protein levels of Oct4 observed during differentiation (Fig. 5a–c), creates an indication for a potential regulation of the Oct4 protein by TRIM32. TRIM32 could be regulating Oct4 protein levels through an ubiquitination-mediated way in a similar manner to its regulation of cMyc. Hence, we investigated whether Oct4 is a potential ubiquitination target of TRIM32.

**Figure 7:**
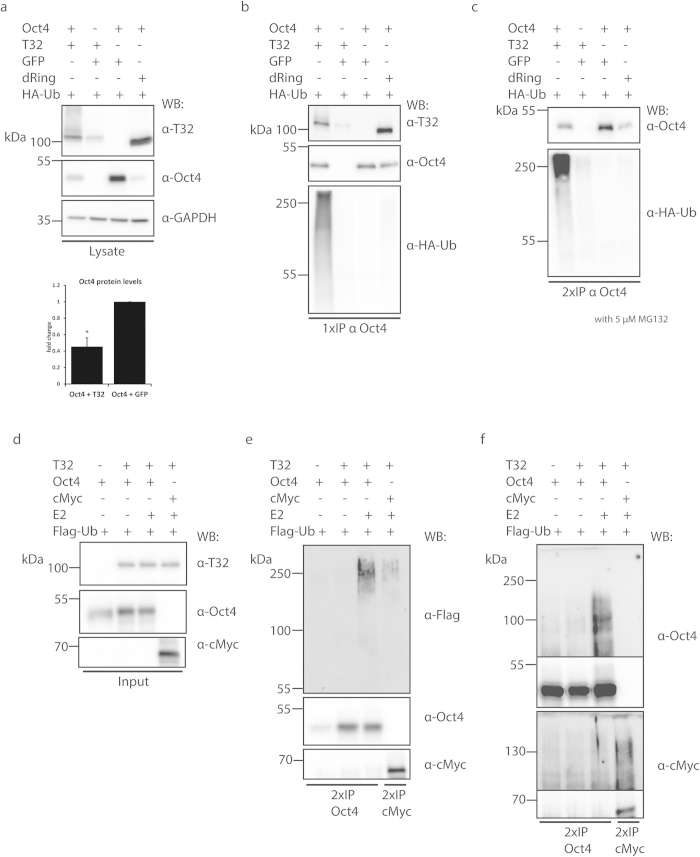
TRIM32 can ubiquitinate Oct4 (**a**) HEK293T cells were transfected as indicated. TRIM32, Oct4 and GAPDH were detected with specific antibodies as indicated for the lysate fraction. Cells were treated with 5 μM MG132 for 6hrs before lysis. Oct4 levels were quantified using Image J software. Values represent normalized averages ± SEM. (**b**) Oct4 was immunoprecipitated once (1xIP) using a specific anti-Oct4 antibody. TRIM32 that associated with Oct4 was detected with an anti-TRIM32 antibody. Ubiquitinated Oct4 was detected using anti-HA antibody directed against the HA-tag of ubiquitin. (**c**) O**c**t4 was immunoprecipitated twice (2xIP) and ubiquitinated Oct4 was detected using anti-HA antibody. (**d–f**) *In vitro* ubiquitination assay using recombinant proteins. Activated E2 (UbcH5a) was incubated with the indicated components followed by 2xIP of Oct4 and cMyc with the specific antibodies. Ubiquitination of substrates was detected with an anti-FLAG antibody directed against the FLAG-tag of ubiquitin and Oct4 or cMyc specific antibodies. The blot in (**d**) shows the input, while (**e,f**) display the 2xIP results. All gels were run under similar experimental conditions. Full-length blots are presented in Supplementary Figure 6.

To explore this possibility we employed an *in vivo* ubiquitination assay using wild-type-Oct4, wild-type-TRIM32 but also a ΔRING-TRIM32 that lacks the RING domain containing the ubiquitin ligase and has therefore no ubiquitination activity. HEK293T cells were transfected with wild-type-Oct4, HA-tagged ubiquitin (HA-Ub) and wild-type-TRIM32 or ΔRING-TRIM32 and protein ubiquitination was analysed after 48hrs. Quantifying Oct4 protein levels in lysates yielded a significant 50% reduction when wild-type-TRIM32 was co-expressed (Fig. 7a and Suppl Figure 6a–c). Notably, the ΔRING-TRIM32 also reduced Oct4 protein levels, which could be an effect mediated by an ubiquitination independent, potentially miRNA dependent, action of TRIM32 on Oct4. To examine whether the reduction observed is due to a direct interaction between TRIM32 and Oct4, an Oct4 specific antibody was used to precipitate it. This resulted in co-precipitation of both wild-type-TRIM32 and ΔRING-TRIM32 (Fig. 7b and Suppl Figure 6d and e) indicating that TRIM32 and Oct4 do indeed interact. Staining the Oct4 Immunoprecipitation (IP) blot for HA-Ub showed a prominent ubiquitination smear only in presence of wild-type-TRIM32, indicating a possible ubiquitination of Oct4 by TRIM32 (Fig. 7b and Suppl Figure 6f). To exclude the possibility that this smear results from contamination by other ubiquitinated proteins that by chance interact with Oct4 and were consequently co-precipitated, Oct4 is precipitated a second time (2xIP) from the first IP ensuring the exclusive isolation of Oct4. In this case, the smear was retained in the lane where wild-type-TRIM32 is co-expressed but not where no TRIM32 or the ΔRING-TRIM32 construct were expressed (Fig. 7c, 1^st^ lane and Suppl Figure 6g and h). Hence TRIM32 can specifically ubiquitinate Oct4 and thereby potentially regulate its degradation via the proteasome system. However, by a yet unknown mechanism TRIM32 lacking the ubiquitin ligase activity (∆RING-TRIM32) is also able to mediate a down-regulation of Oct4 levels (see discussion).

To further support these data, an *in vitro* ubiquitination assay, using purified recombinant proteins, was conducted. Recombinant cMyc protein was used as a positive control as it is one of TRIM32’s validated ubiquitination targets^32^ (Fig. 7d and Suppl Figure 6i–k). Similarly to the *in vivo* data, precipitating Oct4 twice resulted in an ubiquitination smear when the blot was stained for FLAG-Ub (Fig. 7e, 3^rd^ lane and Suppl Figure 6m) and Oct4 or c-Myc (Fig. 7f, Suppl Figure 6l–r). This further supports that TRIM32 and Oct4 do physically interact, so that TRIM32 can specifically ubiquitinate Oct4 and thereby target it for degradation.

To further support the specificity of the interaction between Oct4 and TRIM32 as well as the IP and ubiquitination assay, we performed additional *in vivo* ubiquitination control experiments co-expressing TRIM32, GFP and HA-Ub (Suppl Figure 7a–c). In that case, TRIM32 could not be precipitated with an anti-GFP antibody (Suppl Figure 7a) and no ubiquitination of GFP could be observed (Suppl Figure 7c). Finally, repeating the *in vivo* ubiquitination experiments with Sox2 instead of Oct4 failed to show any ubiquitination specific smears (Suppl Figure 7d, e) after the 2xIP of Sox2 (Suppl Figure 7d–j). All in all this supports the specificity of the interaction between TRIM32 and Oct4.

## Discussion

Pluripotent stem cells provide a great tool not only for regenerative therapies but also for *in vitro* disease modelling. The development of the iPSC technology in 2006 has enabled this field of research to circumvent several of the ethical issues associated with the use of human ESCs^1^,^47^. Yet the implementation of these cells in therapy is subject to the limited knowledge on the exact molecular mechanisms controlling their generation and maintenance. Additionally, the inefficiency of various differentiation protocols to generate pure populations of the exact required cell types poses further challenges to the field. Hence, analysing the roles of single factors involved in these processes will increase the potential for their use in therapeutic approaches^48^. Here we show that the cell fate determinant TRIM32 plays an important role in regulating the generation of iPSCs as well as their differentiation via modulating the levels of the key pluripotency factor Oct4.

TRIM32 is expressed in the developing mouse blastocyst as well as in mESCs. Obviously, it is not required for the maintenance of these cells since TRIM32-ko iPSCs could be generated and propagated in a similar manner to TRIM32-wt iPSCs. However, TRIM32 levels are low in these cells and additionally it probably is inactive or inhibited. It has been previously shown that TRIM32 interacts with protein kinase ζ (PKCζ) in neural stem cells and that this interaction inhibits TRIM32 from inducing neuronal differentiation^32^. Nonetheless, TRIM32 and PKCζ do not co-localize in mESCs (data not shown) excluding inhibition by PKCζ, but not necessarily by other unknown factors.

It has also been suggested that the auto-ubiquitination status of TRIM32 regulates its cellular localization and thereby its function^32^. We do observe a change in TRIM32’s cellular distribution upon induction of differentiation from being confined to the nucleus to being expressed in both the nucleus and the cytoplasm during differentiation. It is plausible that TRIM32 is shuttled between the cytoplasm and the nucleus to be able to perform the different functions. Two scenarios are conceivable, in the first one, at initiation of differentiation, Oct4 would be ubiquitinated by TRIM32 in the nucleus and then transported to the cytoplasm for degradation. In a second scenario, induction of differentiation would first lead to Oct4 translocation to the cytoplasm where it would be ubiquitinated by TRIM32. Probably TRIM32 can ubiquitinate Oct4 only in the cytoplasm, but not in the nucleus. In any case indeed Oct4 has been shown to be a nucleo-cytoplasmic shuttling protein^49^,^50^.

Alternatively, TRIM32 could indirectly regulate Oct4, since we also observe a reduction in Oct4 levels in presence of the ubiquitin ligase activity lacking ΔRING form of TRIM32. It is conceivable that such an indirect regulation occurs, for instance, through the PIASy protein. TRIM32 has been shown to interact with PIASy to mediate its ubiquitination and degradation in keratinocytes^51^. Within a different context, PIASy interacts with Oct4 to repress its transcriptional activity^52^. Hence, it is plausible that PIASy is stabilized upon expression of TRIM32 ΔRING which in turn inhibits Oct4. Furthermore, since TRIM32 has the ability to activate micro-RNAs^30^, a TRIM32 mediated micro-RNA dependent down-regulation of Oct4 seems to be conceivable^53^,^54^.

Interestingly, TRIM32 modulates both the generation as well as the properly timed differentiation of iPSCs. Hence, it might not be required for pluripotency maintenance but rather for efficient cell fate transitions. The observation that TRIM32 appears in body-like structures in the nuclei of pluripotent stem cells hints at a way to sequester TRIM32 and inhibit its function. It has been previously demonstrated that TRIM32 interacts with 14-3-3 proteins, which sequester it to cytoplasmic bodies and inhibit its auto-ubiquitination and trans-ubiquitination abilities^55^. It is noteworthy that 14-3-3 proteins are important for the proper long-term reconstitution of hematopoietic stem and progenitor cells, probably through an effect of 14-3-3 proteins on the self-renewal of these cells^56^. Whether 14-3-3 proteins regulate the activity of TRIM32 in pluripotent cells remains to be investigated.

The reprogramming potential of MEFs into iPSCs is most efficient when both Oct4 and cMyc are present at higher levels than Sox2 and Klf4^57^. TRIM32 could be decreasing the efficiency of iPSC colony generation through its negative regulatory effect on Oct4 and cMyc. Hence, in TRIM32 deficient MEFs, the protein turnover of cMyc and Oct4 is slowed down leading to the presence of these factors in higher levels in comparison to the TRIM32-wt MEFs and thereby the TRIM32-ko cells yield more colonies.

Another ubiquitin ligase, Wwp2, has been described to regulate Oct4 levels during iPSC differentiation in a concentration dependent manner^58^,^59^. It does not affect Oct4 levels during stem cell maintenance, but it is necessary for the proper differentiation of these cells^60^. Actually, the correct level of Oct4 is extremely important for the fate of cells. Both the increase as well as the decrease in Oct4 levels in ESCs can direct their differentiation into different fates^61^,^62^,^63^,^64^, thus, it is essential that its levels are kept within the proper concentration to maintain pluripotency and avoid spontaneous differentiation. Another ubiquitin ligase, Itch, is required for somatic cell reprogramming and pluripotency maintenance, also by regulating Oct4 ubiquitination^65^. It therefore cannot be excluded that TRIM32 is regulating Oct4 protein turnover under pluripotency maintenance conditions. It was shown that TRIM6, another TRIM family member, acts as an ubiquitin ligase for cMyc without altering its levels in mESCs. However, TRIM6 thereby regulates the transcriptional activity of Oct4^66^. Nevertheless, it remains to be determined, if TRIM32 has an effect on the transcriptional activity of Oct4 under pluripotency conditions.

Supportive of the concept that TRIM32 is mainly essential at cellular transitions is the increase in its expression level when mESCs differentiate. When it is absent the TRIM32-ko iPSCs show perturbed differentiation kinetics, which is also reflected by the differential expression analysis. In the differentiating cells, the number of differentially expressed genes between TRIM32-wt and TRIM32-ko cells increased from the iPSCs stage to 12 days EBs. Despite their perturbed differentiation, TRIM32-ko iPSCs are able to differentiate into cells of all germ layers. Hence, the loss of TRIM32 does not inhibit differentiation completely, but TRIM32 appears to be important for the coordination of an adequately timed differentiation procedure.

The Boolean modelling of the GRNs during differentiation suggests that TRIM32 exerts its function via modulation of Oct4. TRIM32-wt cells show a regular *Oct4* centred network of differentiation while in the absence of TRIM32, this regulatory effect on Oct4 is absent and consequently the differentiation is compensated by a *TRIM28* assisted *E2F1* regulation. TRIM28, also known as TIF1β, has been shown to be essential for embryonic development^67^, but also for the proper differentiation of pluripotent cells^36^,^68^. It can form a complex with Oct4^36^ and binds half of the promoter sites bound by Oct4^69^. Additionally, its non-phosphorylated form is essential for proper differentiation of stem cells^36^. Hence, it is likely that during differentiation of TRIM32-ko cells, TRIM28 acquires a hierarchically higher function to compensate for the increased Oct4 protein levels.

In conclusion, our data show that TRIM32 regulates the pluripotency factor Oct4. Therefore, the absence of TRIM32 increases the efficiency of reprogramming into pluripotency. In addition, the deficiency of TRIM32 alters the pathways taken by pluripotent stem cells when undergoing differentiation. Based on these results we conclude that TRIM32 is a modulator of entry into- as well as exit from pluripotency.

## Materials and Methods

### mESCs culture and differentiation

Two mouse embryonic stem cell lines were used throughout this study (see Figure legends for detailed information). First, C57BL/6 mESCs that were was purchased from Gibco (GIBCO^®^ Mouse (C57BL/6) Embryonic Stem Cells Cat. No. S1503-100) and cultured on irradiated mouse embryonic fibroblasts (Amsbio) in Knockout-DMEM (Invitrogen) supplemented with 15% knockout-serum replacement, 2 mM L-Glutamine (PAA), 2 mM Penicillin Streptomycin (PAA), 0.1 mM non-essential amino acids (Invitrogen), 0.1 mM 2-mercaptoethanol (Invitrogen) and 0.01 μg/ml leukemia inhibitory factor (Millipore). For embryonic body differentiation cells were grown as floating spheres in non-tissue culture dishes in ESCs media without LIF. For immunostaining, they were seeded on gelatin-coated coverslips for the required time.

Second is an E14tg2α feeder free growing mESC line that was generously obtained from Carmen Marin (University Leon) and cultured on 0.1% gelatin in GMEM (Sigma) supplemented with 10% mESCs qualified FBS (Invitrogen), 2 mM L-Glutamine (Gibco), 2 mM Penicillin Streptomycin (PAA), 0.1 mM Non-essential amino acids (Invitrogen), 1 mM Sodium pruvate (Gibco), 0.1 mM 2-Mercaptoethanol (Invitrogen) and 500 U/ml leukemia inhibitory factor (ESGRO Millipore). For embryonic body differentiation cells were grown as floating spheres in non-tissue culture dishes in ESCs media without LIF, but with 15% FBS and 0.05% 2-Mercaptoethanol.

### Immunofluorescence stainings

For immunofluorescence stainings the cells were fixed using 4% paraformaldehyde in 120 mM phosphate buffer, pH 7.4 (PBS) for 15 min followed by permeabilization using 0.05%–0.5% Triton X-100 in PBS depending on the cell type. Cells were blocked in 10% fetal bovine serum in PBS and incubated with 1ry Ab at 4 °C overnight. On the following day, the cells were washed and incubated with the 2ry Ab.

Images were collected with a Zeiss confocal LSM 710 or Zeiss Inverted epifluorescence Observer.Z1 microscope. Image analysis was conducted using Zen software (Zeiss), Adobe Photoshop (Adobe) and Image J.

### Mouse blastocysts

Wild-type mouse blastocysts were generously provided by Michele Boiani (Max Planck Institute for Molecular Biomedicine, Münster) and were fixed in 4% paraformaldehyde in 120 mM phosphate buffer, pH 7.4 (PBS) for 15 min followed by permeabilization using 0.1% Triton X-100 in PBS. Immunofluorescent staining was performed as described above.

### RNA isolation, cDNA and RT_qPCR

RNA was isolated using RNEasy kit (Qiagen) or miRNeasy kit (Qiagen) following manufacturer’s instructions. cDNA synthesis was performed using “SuperScript II Reverse Transcriptase” (Invitrogen) and RT-qPCR was done using “Maxima SYBR Green qPCR Master Mix” (Thermo Fisher) and Roche LightCycler^®^480.

The primers used are listed in supplementary table 1.

### Animals, MEFs and iPSCs generation

TRIM32 knock-out and wild-type mice^31^ were maintained and treated according to approved protocols and in accordance with institutional and national guidelines and regulations (Authorities: Landesamt fuer Natur, Umwelt und Verbraucherschutz Nordrhein-Westfalen).

TRIM32 mouse embryonic fibroblasts were prepared from day 12 old embryos as described elsewhere^1^. A pair of TRIM32-wt and TRIM32-ko MEFs was always generated from the same embryo litter, and in total four different pairs were generated from four different litters.

TRIM32-wt and TRIM32-ko iPSCs were generated as previously described^1^ with some modifications. MEFs were seeded at 5 × 10^4^ cells per well and transduced with four retroviruses encoding each for Oct4, Sox2, cMyc and Klf4. At days 15–20 colonies were picked and expanded on irradiated MEFs. Two pairs of TRIM32-wt and TRIM32-ko clones, from two independent litters, were further expanded and analysed in more detail. The TRIM32-wt and TRIM32-ko iPSCs were cultured in Knockout-DMEM (Invitrogen) supplemented with 16% knockout-serum replacement, 8% mESCs qualified FBS (Invitrogen), 2 mM L-Glutamine (PAA), 2 mM Penicillin Streptomycin (PAA), 0.1 mM Non-essential amino acids (Invitrogen), 0.1 mM 2-Mercaptoethanol (Invitrogen) and 0.01 μg/ml leukemia inhibitory factor (ActiveBiosciences). Differentiation into embryonic bodies was performed using the same media without LIF and with/without 5 μM retinoic acid (Sigma).

### Alkaline phosphatase and flow cytometry

Four pairs of TRIM32-wt and TRIM32-ko MEFs were simultaneously reprogrammed using the protocol described above for the generation of iPSCs, except that here one Lentiviral OSKM construct was used. Reprogramming efficiency was analyzed by alkaline phosphatase staining (Millipore) at day 11 according to manufacturer’s instructions.

For flow cytometric analysis, reprogrammed MEFs were collected via trypsinization, counted and 1 × 10^6^ cells were used per staining. Cells were washed in staining buffer (2% FBS, 0.1% NaN_3_ in 1x PBS) and 15 μl SSEA1-FITC coupled antibody (BD Biosciences) was added for 45 min at 4 °C, followed by the addition of 0.4 μl of Hoechst 33258 (Invitrogen) for 15 min in 100 μl volume. Unlabelled cells were used as a negative control. Flow cytometric analysis was performed using BD Fortessa Cell Analyser.

### Deep RNA sequencing/next generation sequencing (NGS)

TRIM32-wt and TRIM32-ko iPSCs were differentiated as described above and total RNA was collected at day 3 and at day 12 and was isolated using the miRNeasy kit (Qiagen). Integrity and concentration was assessed using Nanodrop 2000C (Thermo Scientific).

A sequencing library for Illumina deep sequencing was constructed using TruSeq RNA Seq kit (version 2) using 200 μg of the total RNA according to the manufacturer’s instructions. Details of this procedures are described elsewhere^70^. Sequencing was conducted on Illumina HiSeq 2500 platform by 36-base-single-end read sequencing. Approximately, 20 million sequences were generated per sample and employed for the analysis. 100 bp-long paired-end RNA-Seq reads were first pre-processed to remove low quality sequences on both the 3′ and 5′ ends using Trimmomatic v0.22^71^ with the following parameters: LEADING:20 TRAILING:20 SLIDINGWINDOW:4:20 MINLEN:50. Pre-processed reads were aligned to the mouse reference genome (Ensembl release 72, GRCh38) using TopHat v1.2.0.6^72^ with parameter -G and known splice junctions from Ensembl (ftp://ftp.ensembl.org/pub/release-72/gtf/mus_musculus/Mus_musculus.GRCm38.72.gtf.gz). Properly paired reads with a mapping quality of at least 20 were extracted from the resulting BAM file using SAMtools^73^ for further analyses. Read counts per gene were calculated for each sample with Htseq-count^74^ with default parameters, following the recommendation of the software developer for RNA-Seq data.

### RNA-Seq Statistical Analysis

Normalization and differential expression analysis of the pre-processed RNA-seq count data were performed using the edgeR and limma packages in Bioconductor/R statistical language. The statistical analysis steps consisted of (i) non-specific filtering of genes, (ii) library normalization, (iii) mean-variance modeling based transformation, (iv) differential expression analysis and (v) an overrepresentation analysis. For the non-specific filtering, genes whose sum of htseq-counts across all 12 samples (i.e., 6 wt and 6 ko, for iPSC, day 3 and day 12, with 3 replicates for each time-point) is less than 20 were removed, to increase detection power. Later, library normalization was performed using the trimmed mean of M-values (TMM) method in edgeR^75^ to adjust for differences in library size. This was done in conjunction with Voom^76^, a mean-variance modeling based transformation of discrete RNA-seq read count data to continuous micro-array like log2-cpm data, and with precision weight design matrices. This is majorly performed, to access the large body of analysis methodologies that are developed for microarray studies to RNA-seq log-count data. Then, normalized expression levels were compared between the sample groups of interest using empirical Bayes methodology of the limma package^77^ and significant differences in expression were identified by adjusted P-values. P-values were corrected for multiple testing using the Benjamini-Hochberg method^78^. The default cut-off used for a statistically significant differentially expressed gene was an adjusted P-value of less than 0.05, unless otherwise stated. Genes expressed differentially between different groups of interest were interpreted using an overrepresentation analysis for GO-terms and KEGG pathways with the RDAVIDWebService^45^ package in Bioconductor/R. This is a web based API for the well-known bioinformatics service DAVID.

### Boolean Modelling of Differential Expression Data

Specific changes in the regulatory mechanisms of iPSCs differentiation to EBs in TRIM32-wt and TRIM32-ko data were elucidated using weighted Boolean modelling of gene regulatory networks (GRN). Detailed methodology for GRN model building between different groups of interest will be presented elsewhere (Perumal, TM and Del Sol, A, unpublished). Briefly, it involves the following steps: (i) gene selection, (ii) GRN inference, (iii) weighted Boolean modelling, (iv) model pruning and (iv) model analysis. These are detailed below:

#### Gene selection

For each group of interest, the top 2000 differentially expressed genes that are not referenced as transcription factors (TFs) and the top 500 differentially expressed TFs were selected for network modelling. TFCheckpoints database^79^ was used to segregate TFs from non-TFs in the global gene expression dataset.

#### GRN inference

Here, GRNs for the above selected set of genes were inferred using an integrative approach, where both the literature based text-mining networks and machine learning based data-inferred networks are combined. For each set of genes and TF, we used MetaCore^80^ software to obtain the most parsimonious GRN possible from literature, using “direct interactions” parameter, and hence showing only functional binding interactions. Data-driven GRNs for the selected 2500 genes were obtained using simple Pearson correlation between genes, CLR^81^ and GENIE3^82^ methodologies. Top 0.01% of the inferred interactions using all three methods were selected for further analysis.

#### Weighted Boolean modelling

Combined GRNs of both the literature and machine learning based methods were used for Boolean modelling. In our Boolean models, the strength of each interaction were not equal, rather they were measured from data using a granger causation metric^83^ based on which the truth table for Boolean simulations was formulated.

#### Model pruning

Since the model was obtained by combining both text mining and data driven knowledge, it is utmost necessary to contextualize the model to the data from the group of interest. To this end, we use a modified version of our contextualization algorithm presented elsewhere^84^. In summary, this algorithm removes inconsistent interactions by matching the simulated data of the model to the Booleanized differential expression data from high-throughput experiments. For each group of interest, 5 sets of 20 different Boolean models were obtained for further analysis. This ensures heterogeneity and robustness in our predictions.

#### Model analysis

Once the desirable Boolean models were obtained, the causal regulatory mechanisms between any two stable expression patterns were elucidated using the differential stability analysis presented elsewhere. In this analysis, differentially expressed positive feedback loops, which are also termed differential stability elements, were detected. These are shown to regulate the cellular transition between states of interest^46^.

### *In vivo* Ubiquitination assay

HEK293T cells were transfected using Turbofect (Fermentas) according to the manufacturer’s instructions. After 48 hrs the cells were lysed with nuclear lysis buffer (50 mM TRIS (pH 7.5), 0.5M NaCl, 1% NP-40, 1% DOC, 0.1% SDS, 2 mM EDTA, and Complete protease inhibitor (Roche) in ddH_2_O) for 30 min at 4 °C and the percentage of SDS was reduced to 0.07% by adding TRIS/EDTA after lysis. For immunoprecipitation the protein concentrations were measured using a BCA protein assay kit (Thermo Scientific) according to the manufacturer’s instructions. Equal protein amounts were incubated with the precipitation antibody at 4 °C for 4 hrs and then with protein-G Sepharose beads (GE Healthcare) overnight. For a 1xIP the bound proteins were eluted in protein sample buffer for 15 min at 95 °C. For a 2xIP the bound proteins were cooked in 1% SDS for 15 min to break all protein-protein interactions. The eluate was used for a second round of immunoprecipitation as described above. Analysis of western blots was done using Adobe Photoshop (Adobe) and Image J software.

### *In vitro* Ubiquitination assay

Recombinant TRIM32 protein was generated using the TNT^®^ T7 Quick Coupled Transcription/Translation System (Promega) following manufacturer’s instructions. Oct4 and cMyc recombinant proteins were obtained from Abcam. *In vitro* ubiquitination assay was performed as previously described^32^. In brief, Oct4 or cMyc were incubated with activated E1 enzyme, E2 enzyme and FLAG-Ub (Boston Biochem) at RT for 1 hr. That was followed by a 2xIP of either Oct4 or cMyc using specific antibodies as described above. Western blots were stained using an anti-FLAG-antibody to detect ubiquitinated proteins.

### Antibodies

The following primary antibodies were used for Immunofluorescence stainings: m anti TRIM32 (Abnova), rb anti TRIM32 (Gramsch Laboratories), m anti-Oct3/4 (Santa Cruz), rb anti-Oct3/4 (Abcam), rb anti-Sox2 (Abcam), rb anti-cMyc (Santa Cruz), rb anti Klf4 (Abcam), rb anti-Nanog (Millipore), m anti SSEA1 (Thermo Scientific), rb anti-doublecortin x (Abcam), rb anti-Lin28 (Abcam), m anti-alpha smooth muscle actin (Abcam), m anti-alpha feto protein (R&D systems), gt anti-Sox1 (R&D systems), rb anti-Pax6 (Covance), m anti-Tuj1 (Covance), m anti-Ki67 (BD Biosciences). As secondary antibodies Alexa-fluorophore conjugated antibodies (Invitrogen) were used and DNA was counterstained using Hoechst 33258 (Invitrogen). For western blot analysis, the following antibodies were additionally used: m anti-Flag (Sigma), m anti-HA (Roche Applied Sciences), rb anti-GAPDH (Abcam), HRP-linked antibodies (GE Healthcare).

## Additional Information

**How to cite this article**: Bahnassawy, L. *et al.* TRIM32 modulates pluripotency entry and exit by directly regulating Oct4 stability. *Sci. Rep.* **5**, 13456; doi: 10.1038/srep13456 (2015).

## Supplementary Material

Supplementary Information
