# Supplementary material for: TRIM32 modulates pluripotency entry and exit by directly regulating Oct4 stability
Source: Sci Rep. 2015 Aug 26;5:13456. doi: 10.1038/srep13456 (PMC4642535; doi:10.1038/srep13456)
Supplement: Supplementary Information [file srep13456-s1.pdf]

TRIM32 modulates pluripotency entry and exit by directly regulating Oct4 stability

- Supplementary Information -

Lamia'a Bahnassawy[1, 2], Thanneer M Perumal[3§], Laura Gonzalez-Cano[2], Anna-Lena Hillje[2], Leila Taher[4], Wojciech Makalowski[5], Yutaka Suzuki[6], Georg Fuellen[4], Antonio del Sol[3], Jens Christian Schwamborn[1, 2 *]

[1]Westfälische Wilhelms-Universität Münster, ZMBE, Institute of Cell Biology, Stem Cell Biology and Regeneration Group, Von-Esmarch-Str. 56, 48149 Münster, Germany

[2]Luxembourg Centre for Systems Biomedicine (LCSB), Developmental and Cellular Biology, University of Luxembourg, 7 avenue des Hauts-Fourneaux, 4362 Esch-Belval, Luxembourg

[3]Luxembourg Centre for Systems Biomedicine (LCSB), Computational Biology, University of Luxembourg, 7 avenue des Hauts-Fourneaux, 4362 Esch-Belval, Luxembourg

[4]Institute for Biostatistics and Informatics in Medicine und Ageing Research, Rostock University Medical Centre, Ernst-Heydemann-Str. 8, 18057 Rostock, Germany

[5]Westfälische Wilhelms-Universität Münster, Institute of Bioinformatics, Niels-Stensen-Straße 14, 48149 Münster, Germany

[6]Department of Medical Genome Sciences, University of Tokyo, 5-1-5 Kashiwanoha, Kashiwa-shi, Chiba-ken 227-8561, Japan.

§ current address: Sage Bionetworks, Seattle, Washington, USA 98109

* Correspondence: jens.schwamborn@uni.lu

Supplementary figure legends

Supplementary figure 1: TRIM32 is expressed in mESCs

a) Immunostainings of C57BL/6 mESCs stained for the indicated markers (upper boxes).

b-f) RT-qPCR for the indicated mRNA in differentiating EBs. EBs were generated in ESC media without LIF and allowed to grow for 12 days. RNA was isolated every 4 days. Represented are averages from at least three independent experiments in arbitrary units (au). Error bars represent SEM. Statistical analysis was performed using t-test or a Mann-Whitney rank sum test (* $p \leq 0.05$, ** $p \leq 0.01$).

Supplementary figure 2: reprogrammed MEFs express SSEA1 after 11 days

a) Western blots from TRIM32-wt and TRIM32-ko MEFs probed for TRIM32 and GAPDH as loading control.

b) Immunostainings of reprogrammed TRIM32-wt and TRIM32-ko MEFs at d11 for the indicated markers (upper boxes). Displayed are maximum intensity projections of the imaged colonies.

c) Western blots from TRIM32-wt and TRIM32-ko iPSCs probed for Oct4, Sox2, Nanog and GAPDH as loading control.

Supplementary figure 3: Basic characterization of TRIM32-wt and TRIM32-ko iPSCs

a) Brightfield images of TRIM32-wt and TRIM32-ko iPSCs growing on MEF feeder layer. Shown are the clones that were used for the downstream analysis.

b) RT-qPCR for the 4 pMX viral plasmids used showing absence of pMX viral plasmids expression in the iPSCs in comparison to MEFs 4 days after viral transduction.

c) Immunostainings of TRIM32-wt and TRIM32-ko iPSCs stained for the indicated markers (upper boxes)

d) Brightfield images of TRIM32-wt and TRIM32-ko 3 day old embryonic bodies grown in absence of LIF.

e) Immunostainings of 14 day long differentiated TRIM32-wt and TRIM32-ko iPSCs. EBs were allowed to grow in ESC media without LIF for 3 days, after which they were seeded on Gelatine and allowed to grow till day 14. Cells were then stained for the indicated markers (upper boxes).

Supplementary figure 4: differentiation of TRIM32-wt and TRIM32-ko iPSCs after 3 and 12 days

a) Brightfield images of TRIM32-wt and TRIM32-ko 3 day old embryonic bodies grown in absence of LIF with the addition of 5µM retinoic acid.

b) Immunostainings of 3 day old embryonic bodies (EBs) for the indicated markers (upper boxes). EBs were generated from either TRIM32-wt iPSCs or TRIM32-ko iPSCs and were grown in ESC media without LIF with 5µM retinoic acid for 3 days after which they were allowed to adhere to Matrigel coated cover slips overnight. Images show 3x3 tile-scans.

c) Immunostainings of 12 day old embryonic bodies (EBs) for the indicated markers (upper boxes). EBs were generated from either TRIM32-wt iPSCs or TRIM32-ko iPSCs and were grown in ESC media without LIF with 5µM retinoic acid for 12 days. They were seeded on Matrigel coated coverslips on day 6. . Images show 3x3 tile-scans.

d-j) quantification of the percentage of positive cells for the indicated markers in b-c after 3 and 12 days of differentiation. Values represent averages from 2 independent clones for both TRIM32-wt and TRIM32-ko cells. At least 3 3x3 tile-scans were quantified per condition i.e. no. of cells counted ≥ 200 per cell line and per condition. Statistical analysis was performed based on the no. of tile-scans quantified using a t-test or a Mann-Whitney rank sum test (*p≤0.05). Displayed are averages in percentage ± SEM.

<u>Supplementary figure 5: Differential expression analysis between TRIM32-wt and TRIM32-ko cells and complete GRNs for TRIM32-wt and TRIM32-ko iPSCs differentiation to 3 days and 12 days EBs</u>

a) *Exploratory clustering of global gene expression.* This figure shows the hierarchal classification tree for log2-cpm values for all 18 normalized samples used in the study.

b) *Overlapping differentially expressed genes.* Venn diagram representing the differential expression between T32 WT and KO at different stages of cellular differentiation (i.e., from iPSCs to 3 day EBs to 12 day EBs).

c) *GRN for TRIM32-wt iPSCs differentiation to 3 day EBs.* Each node represents a gene/protein of interest and each interaction represents an activation or inhibition that are either inferred from literature or obtained from expression data. Each node is divided in two halves and colored. The left and right hand partitions represent expression patterns in iPSCs and 3 day EBs respectively, and the color codes green or red represent down or up regulation of expression. If the node is colored blue, then it is either not significantly expressed for a FDR of 5% or mis-inferred in our model, and hence not used in our analysis. These rules apply to all the following networks.

d) *GRN for TRIM32-ko iPSC differentiation to 3 day EBs.*

e) *GRN for TRIM32-wt iPSC differentiation to 12 day EBs.*

f) *GRN for TRIM32-ko iPSC differentiation to 12 day EBs.*

<u>Supplementary figure 6: full length blots from figure 7</u>

a-h) Full length blots from Oct4 *in vivo* ubiquitination assay (figure 7 a-c)

a-c) whole cell lysates probed for Oct4, TRIM32 and cMyc respectively (figure 7 a)

d-f) 1xIP of Oct4 probed for Oct4, TRIM32 and HA-Ub respectively (figure 7 b)

g-h) 2xIP of Oct4 probed for Oct4 and HA-Ub respectively (figure 7 c)

i-r) Full length blots from *in vitro* ubiquitination assay (figure 7 d-f)

i-k) input probed for TRIM32, Oct4 and cMyc, respectively (figure 7 d)

l-n) 2xIP of Oct4 or cMyc probed for Oct4, Flag-Ub and cMyc respectively (figure 7 e)

o-p) 2xIP of Oct4 or cMyc probed for Oct4 (figure 7 f). o and p represent the same blot analysed using different signal enhancement to visualize the ubiquitination smear

q-r) 2xIP of Oct4 or cMyc probed for cMyc (figure 7 f). q and r represent the same blot analysed using different signal enhancement to visualize the ubiquitination smear.

Supplementary figure 7: GFP control immunoprecipitations, *in vivo* ubiquitination assays for Sox2

a-c) HEK293T cells were transfected with TRIM32, GFP and HA-Ub. GFP was precipitated 1x and 2x and western blots were probed for TRIM32, GFP and HA-Ub respectively. Cells were treated with 5µM MG132 for 6hrs before lysis.

d-e) Sox2 was immunoprecipitated twice using a specific anti-Sox2 antibody. Ubiquitinated Sox2 was not detectable using anti-HA antibody directed against the HA-tag of ubiquitin.

f-j) full length blots from Sox2 *in vivo* ubiquitination assay (supplementary figure 7_1 d-e)

f-h) whole cell lysates probed for Sox2, TRIM32 and GAPDH respectively (supplementary figure 7_1 d)

i-j) 2xIP of Sox2 probed for Sox2 and HA-Ub respectively (supplementary figure 7_1 e).

Supplementary Table

Table 1: Primers used for RT-qPCR

m-cMyc-s	GTTGGAAACCCCGCAGACAG
m-cMyc-as	AATAGGGCTGTACGGAGTCG
m-Klf4-s	GCAGTCACAAGTCCCCTCTC
m-Klf4-as	TAGTCACAAGTGTGGGTGGC
m-Nanog- s	GCATCGAATTCTGGGAACGC
m-Nanog-as	GTCTTCAGAGGAAGGGCGAG
m-Oct4-s	ATTGAGAACCGTGTGAGG
m-Oct4-as	ATTGGCGATGTGAGTGAT
m-Sox2-s	CCCGGACCGCGTCAAGA
m-Sox2-as	CGGACAAAAGTTTCCACTCCG
m-Trim32-s	GCATCCAGGAAGAGCTAG
m-Trim32-as	CTCTACCACTTGACTGTTG
m-GAPDH-s	CTTTGGCATTGTGGAAGGGC
m-GAPDH-as	TGCAGGGATGATGTTCTGGG
m-AFP-s	CTTCCCTCATCCTCCTGCTAC
m-AFP-as	ACAAACTGGGTAAAGGTGATGG
m-SMA-s	GTCCCAGACATCAGGGAGTAA
m-SMAas	TCGGATACTTCAGCGTCAGGA
m-Pax6-s	TACCAGTGTCTACCAGCCAAT
m-Pax6-as	TGCACGAGTATGAGGAGGTCT
m-Nestin-s	AGAGTCAGATCGCTCAGATCC
m-Nestin-as	GCAGAGTCCTGTATGTAGCCAC
m-Dcx-s	AAACTGGAAACCGGAGTTGTC
m-Dcx-as	CGTCTTGGTCGTTACCTGAGT
m-Tuj1-s	TAGACCCCAGCGGCAACTAT
m-Tuj1-as	GTTCCAGGTTCCAAGTCCACC
pMX-Oct4-s	GATCCCAGTGTGGTGGTACGG
pMX-Oct4-as	GGCGAAGTCTGAAGCCAGGT
pMX-Sox2-s	GATCCCAGTGTGGTGGTACGG
pMX-Sox2-as	GGCTTCAGCTCCGTCTCCAT
pMX-Myc-s	GATCCCAGTGTGGTGGTACGG
pMX-Myc-as	TCGAGGTCATAGTTCCTGTTGGTG
pMX-Klf4-s	GATCCCAGTGTGGTGGTACGG
pMX-Klf4-as	GTGGAGAAGGACGGGAGCAG

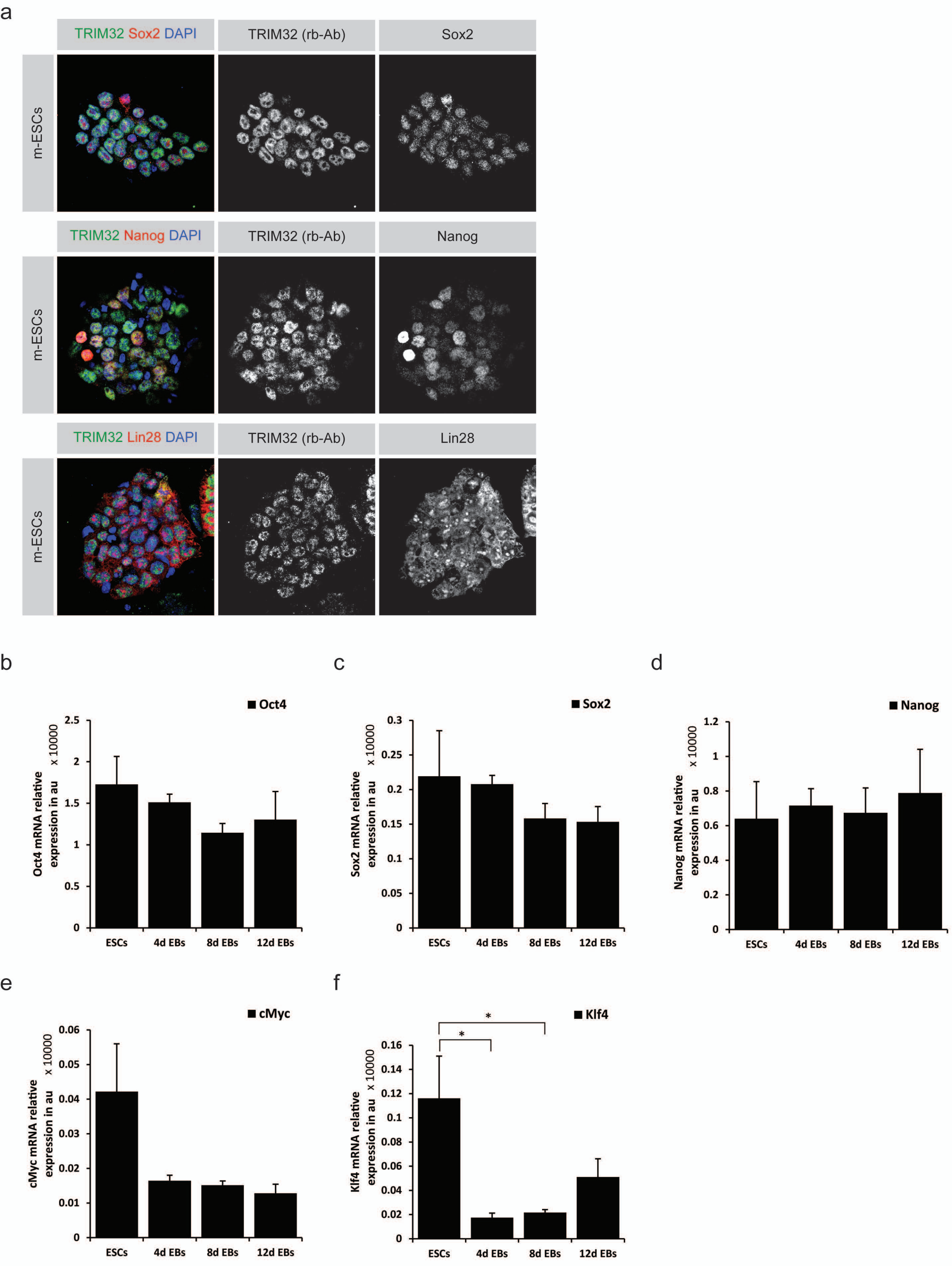

Supplementary Figure 1

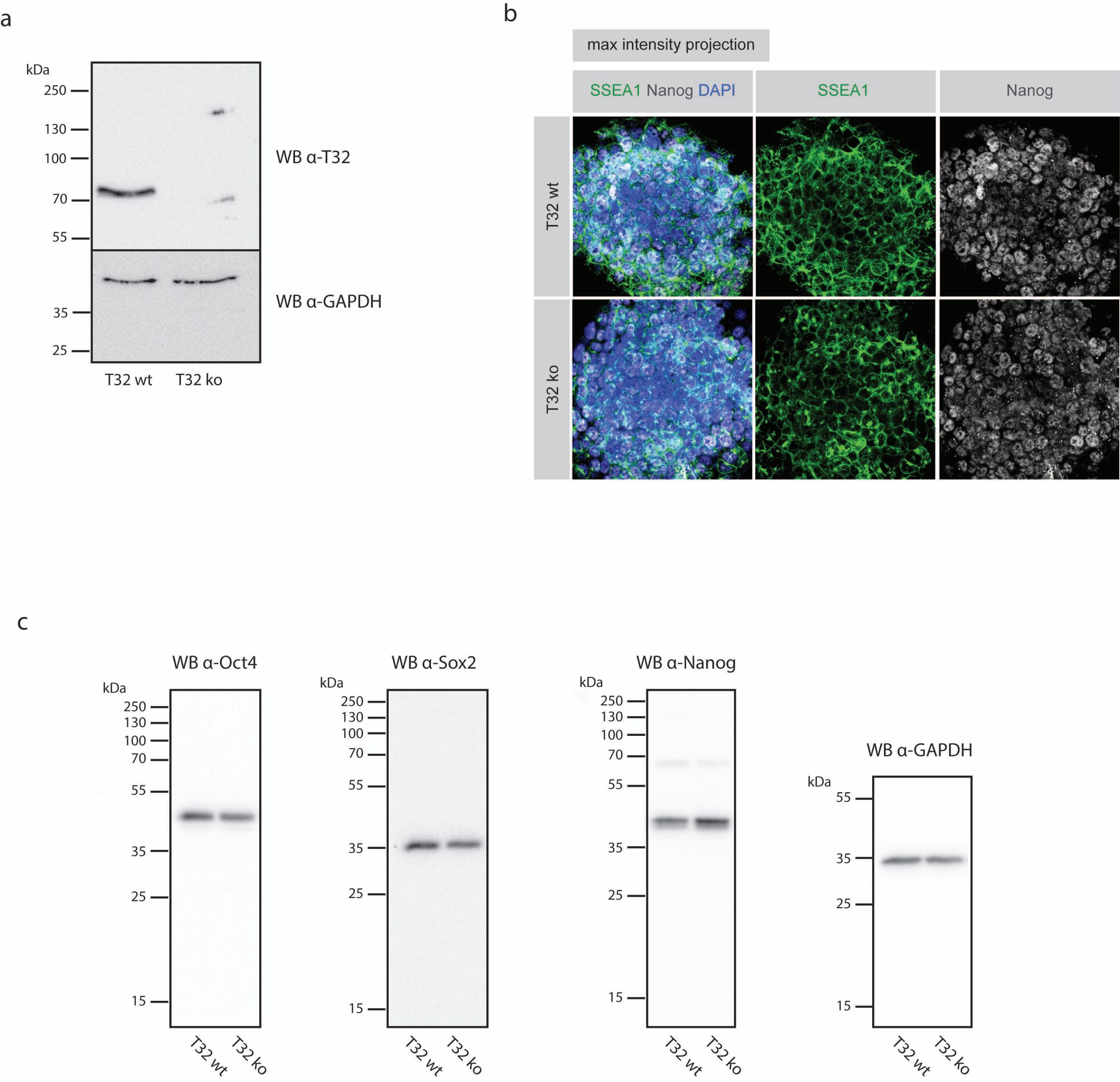

Supplementary Figure 2

a

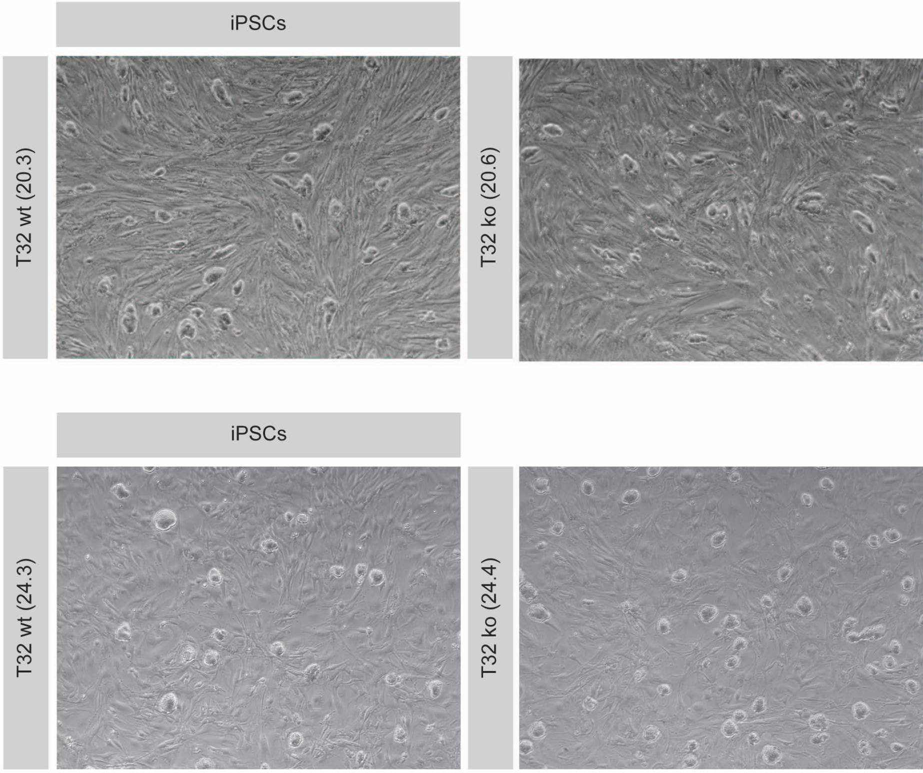

b

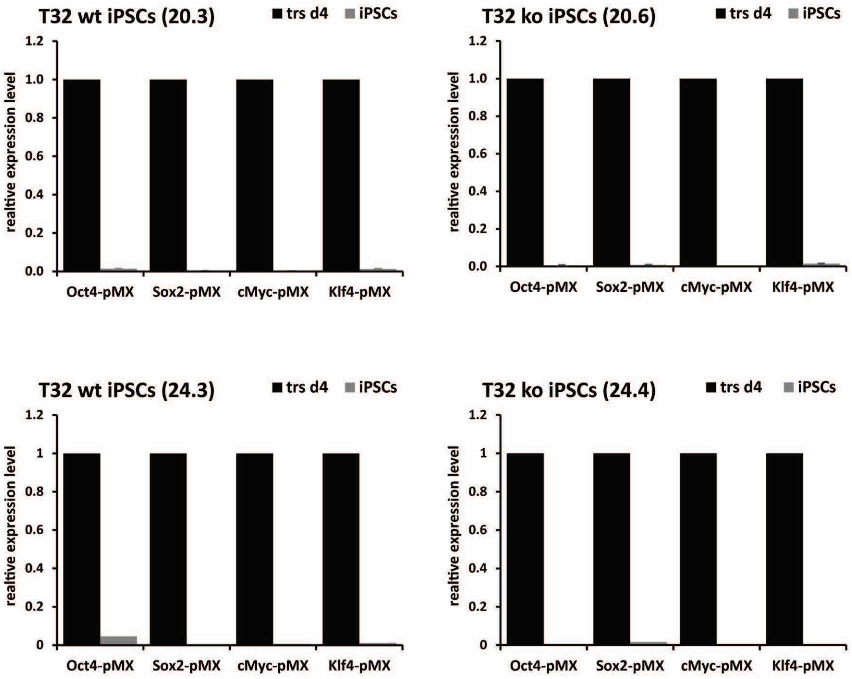

Supplementary Figure 3_1

c

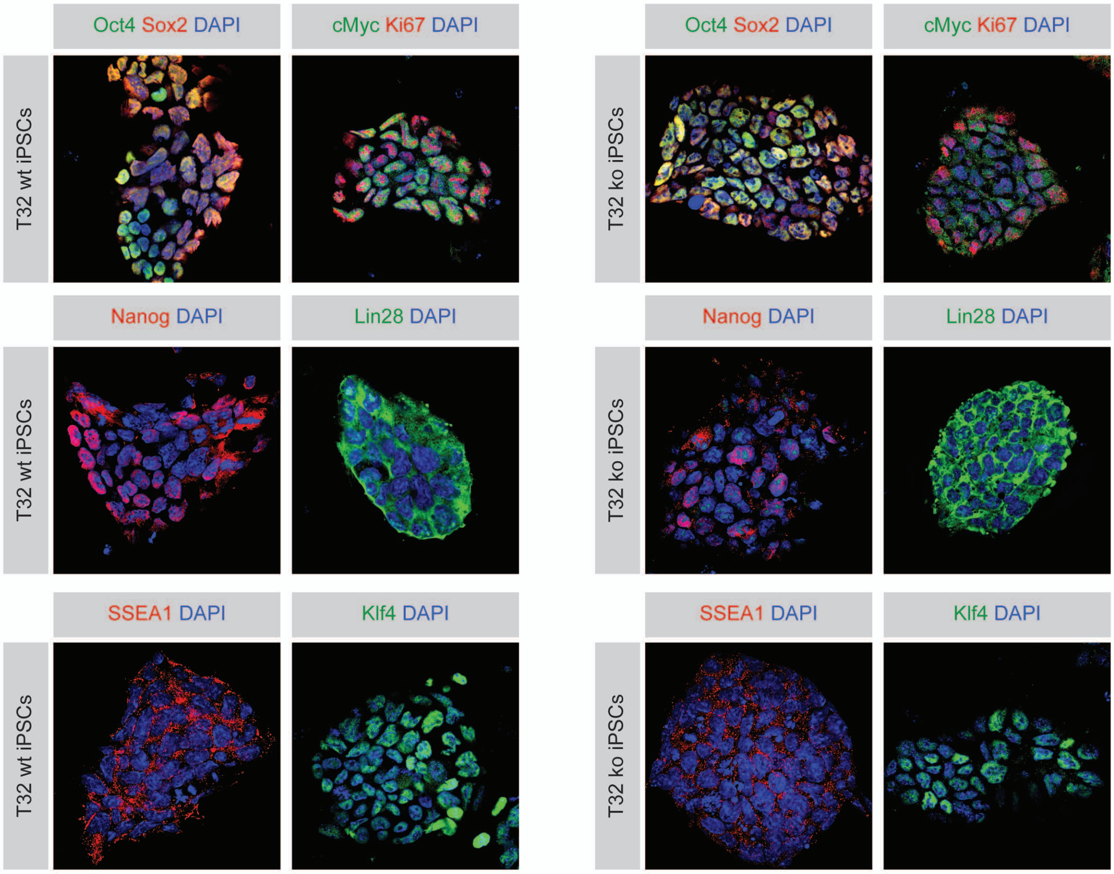

d

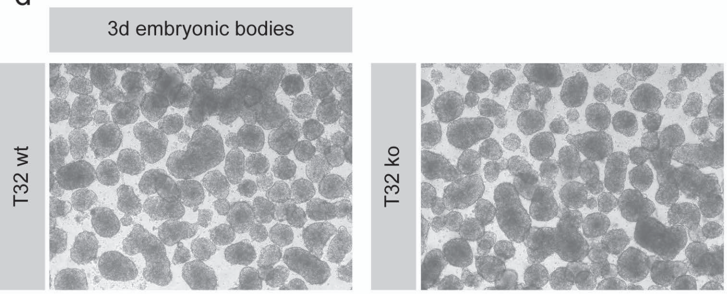

e

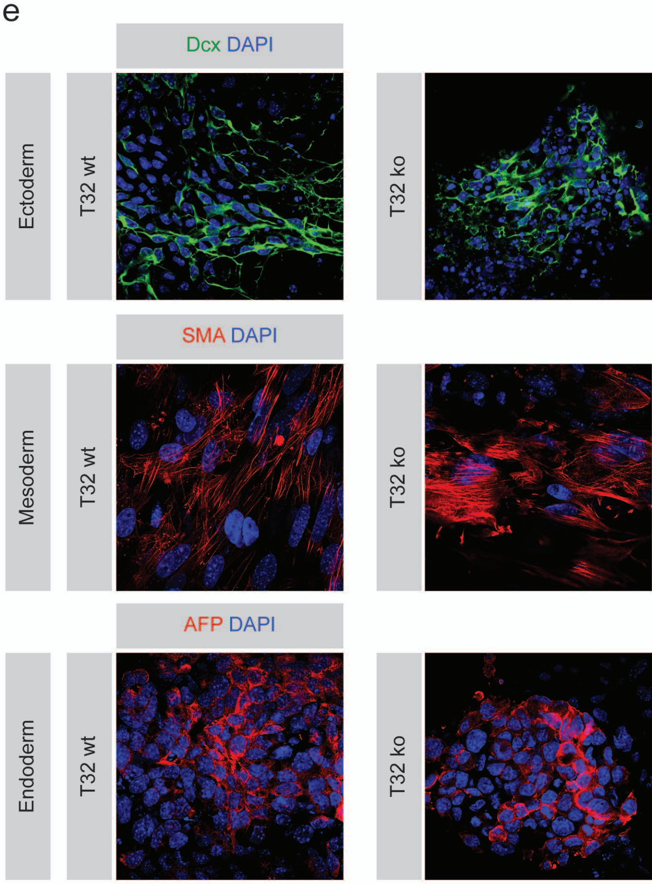

Supplementary Figure 3_2

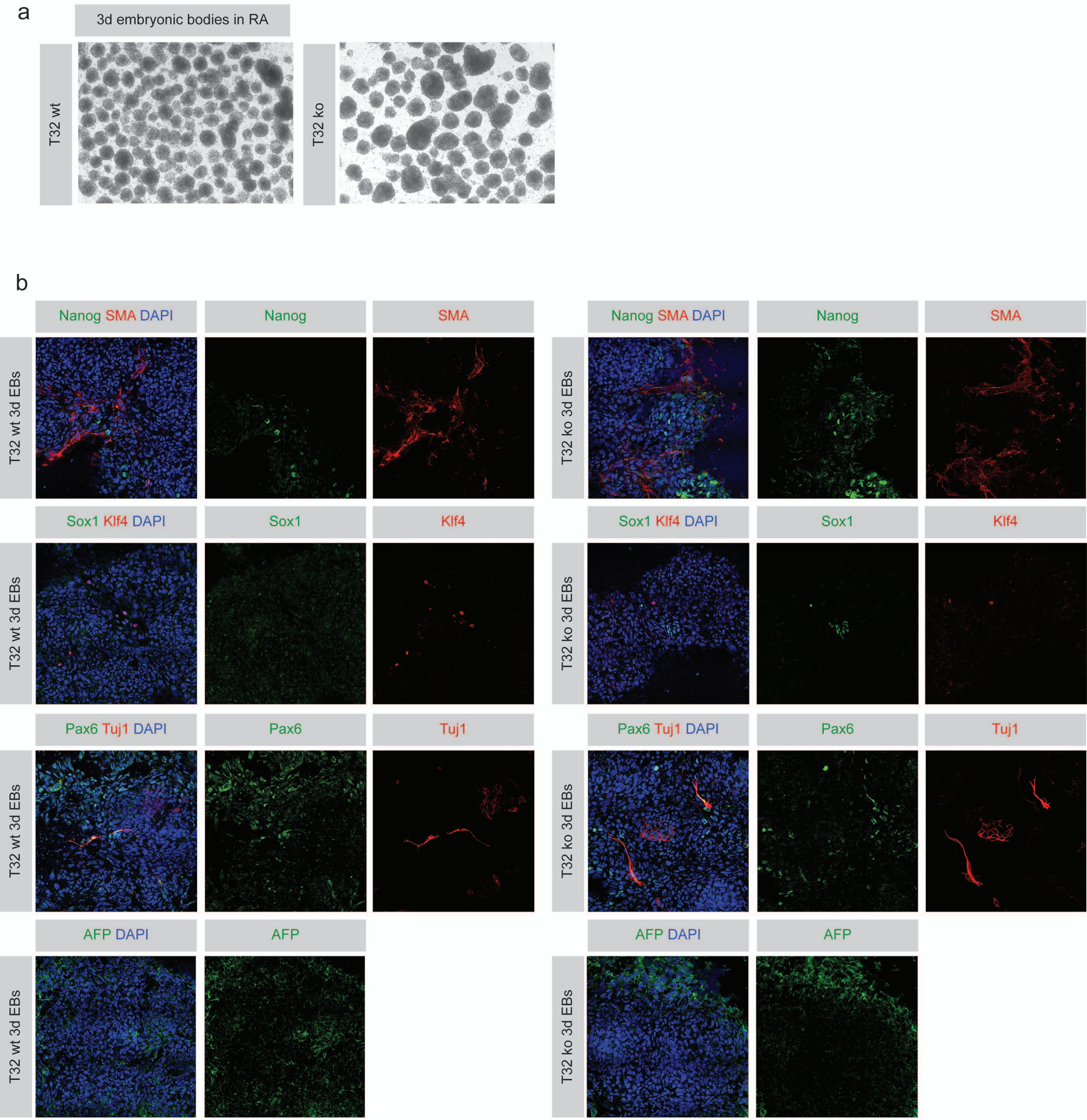

Supplementary Figure 4_1

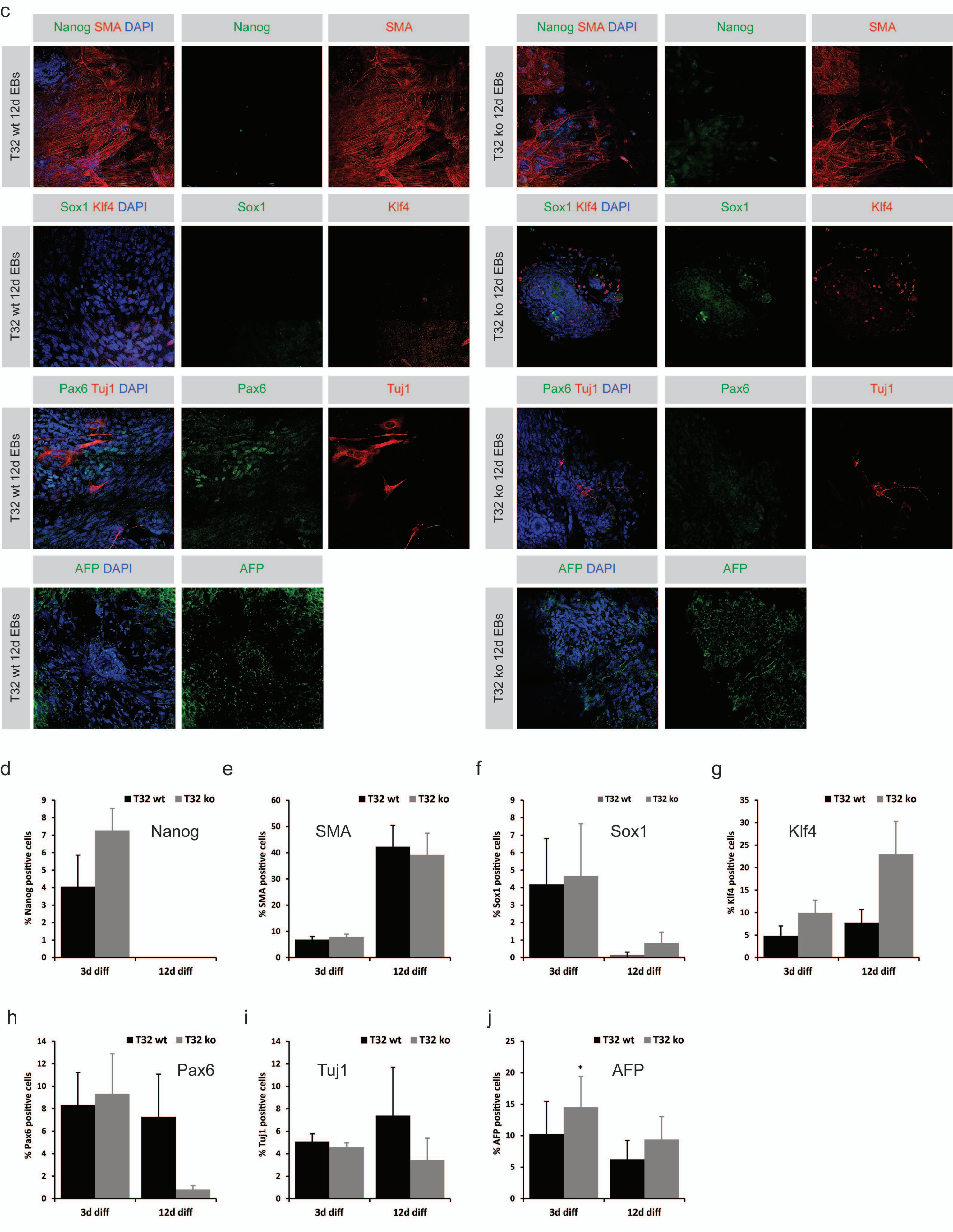

Supplementary Figure 4_2

a

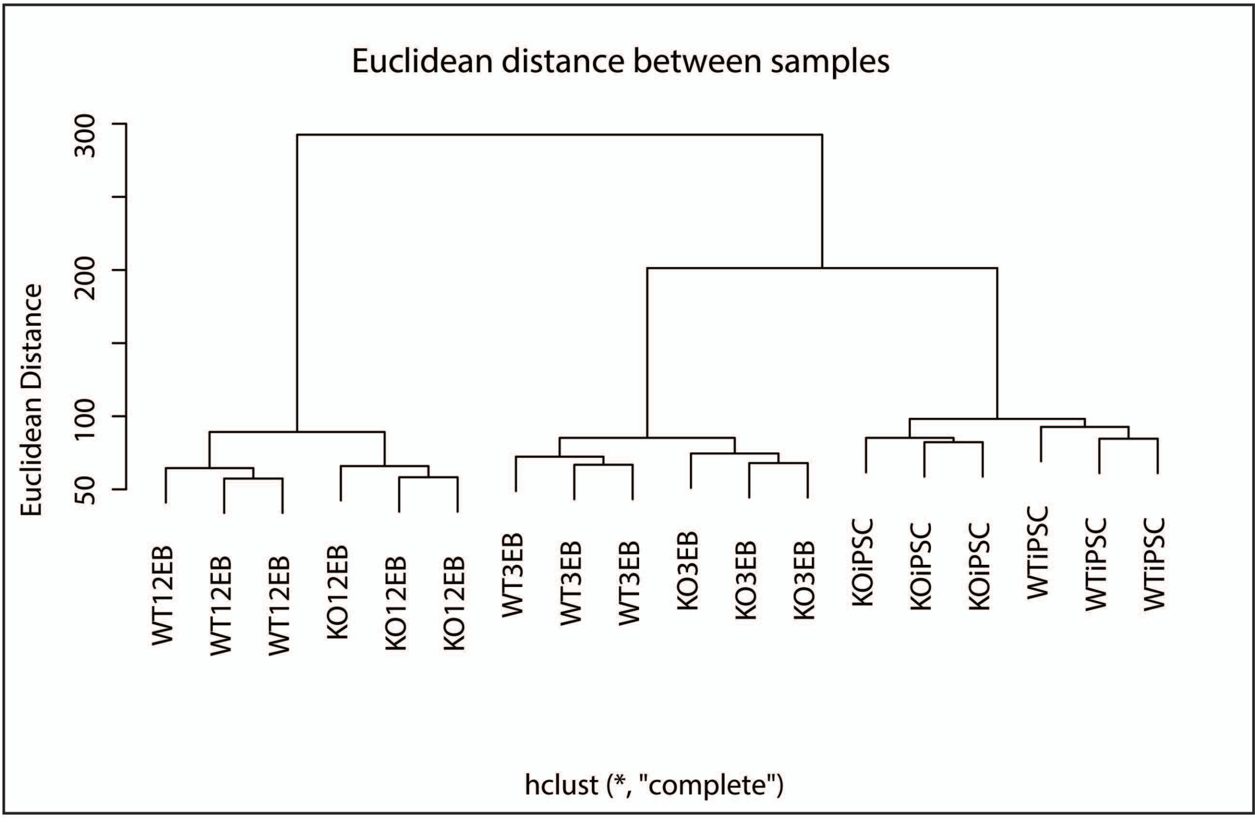

b

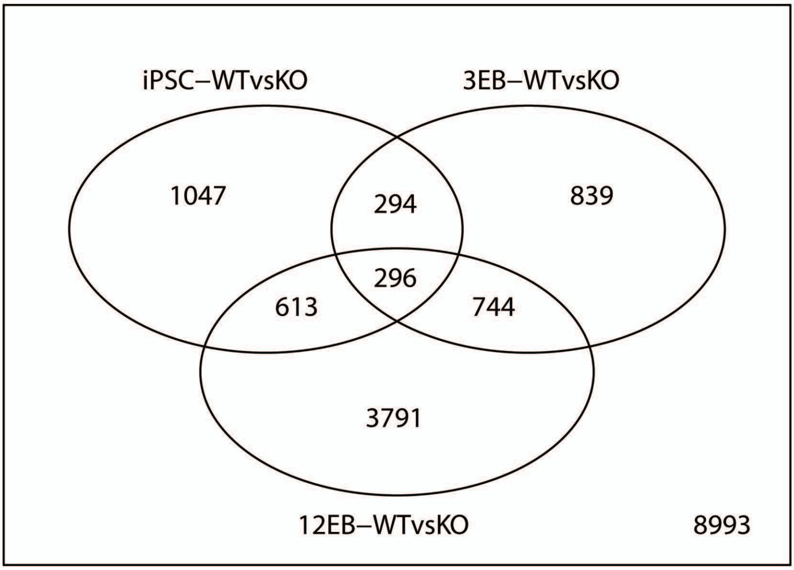

Supplementary Figure 5_1

a

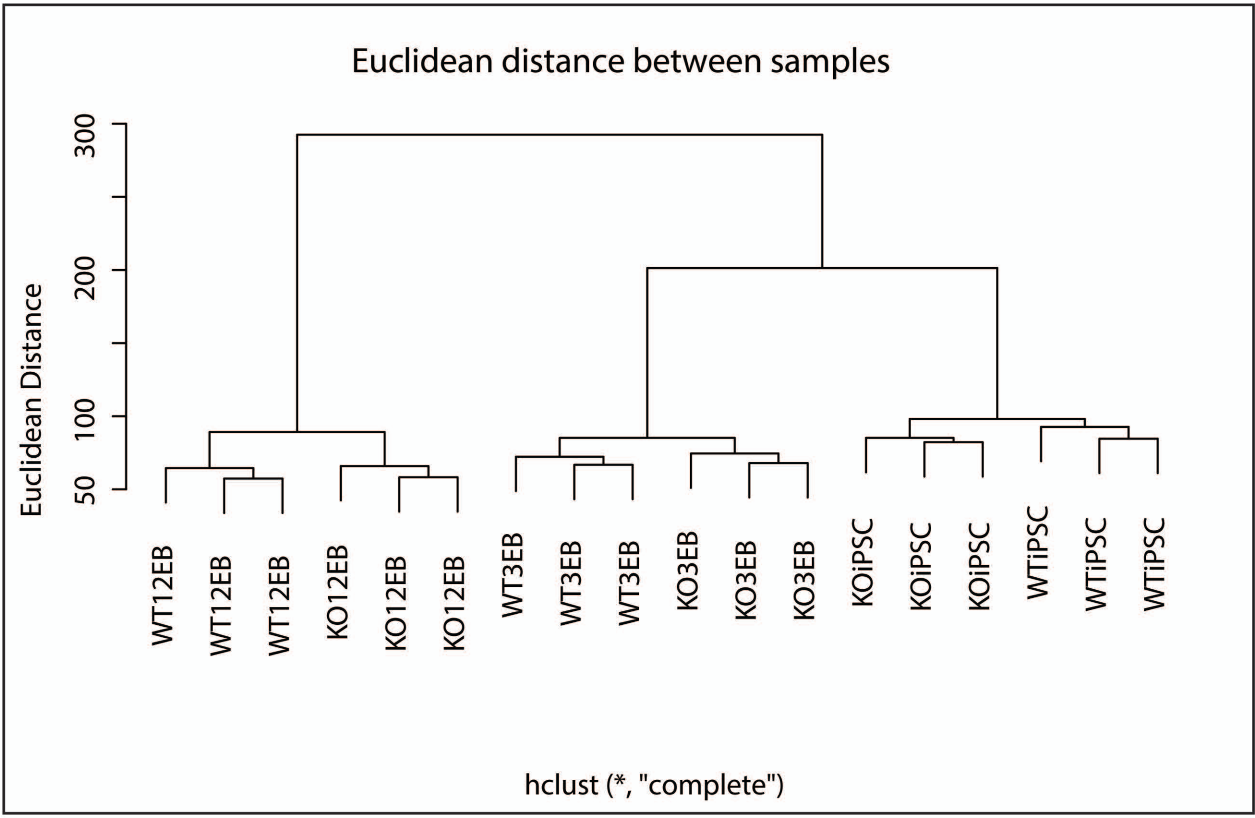

b

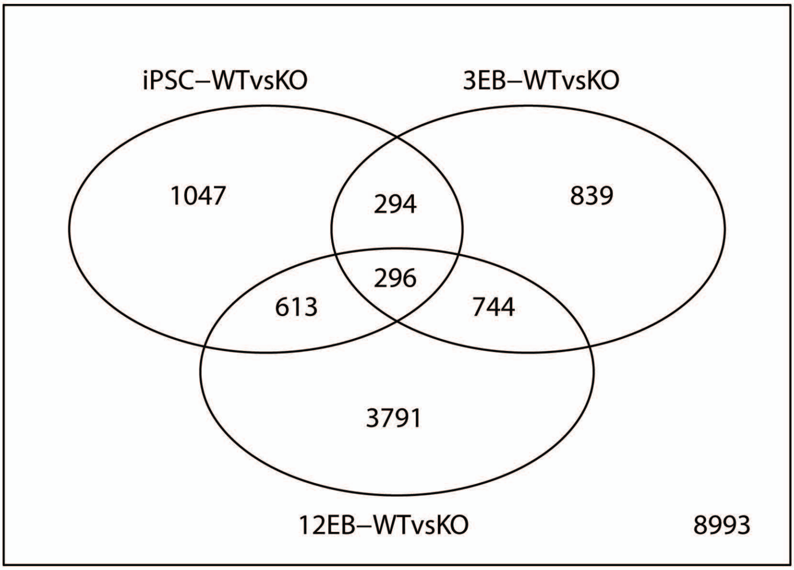

Supplementary Figure 5_1

c

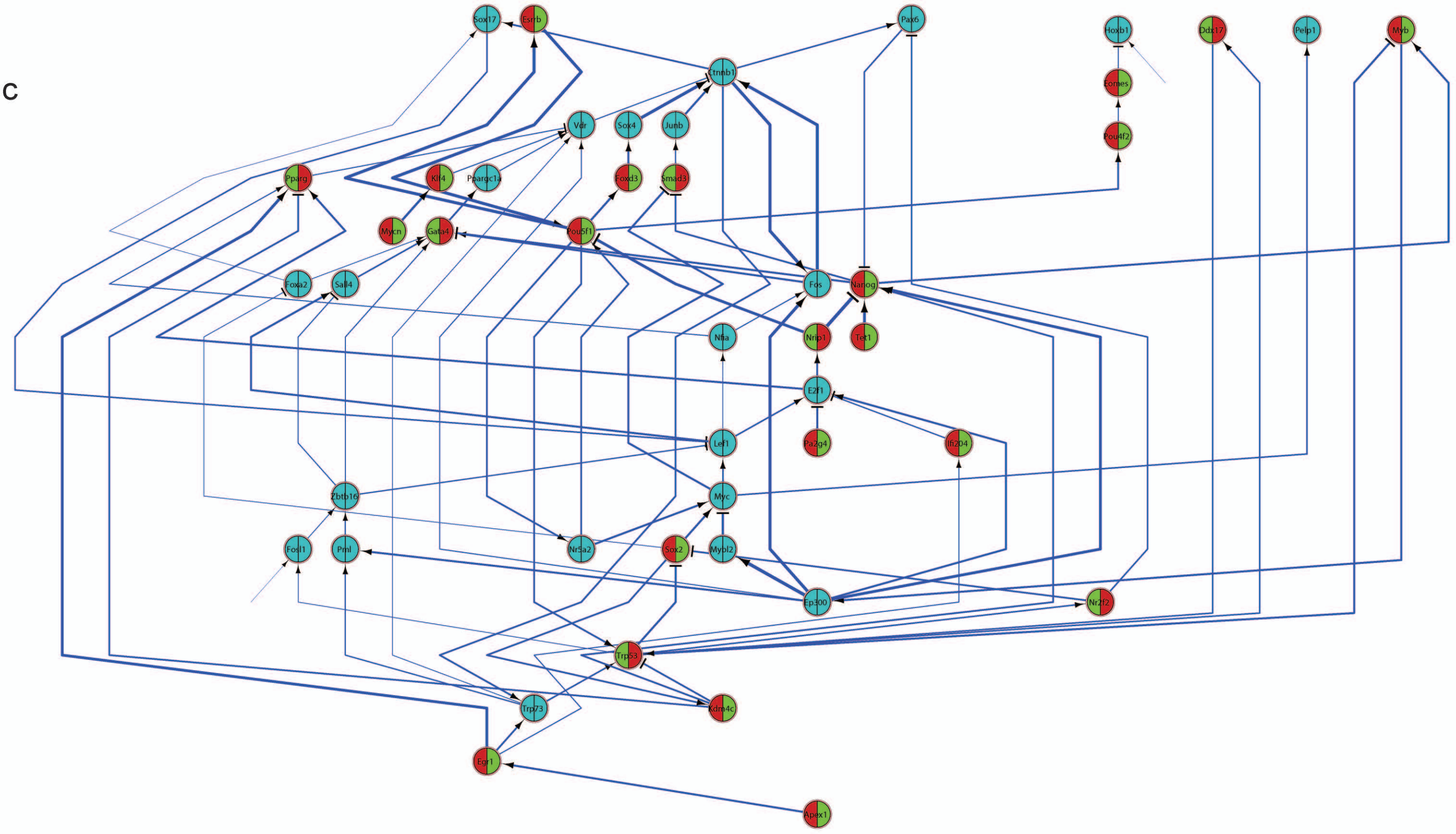

d

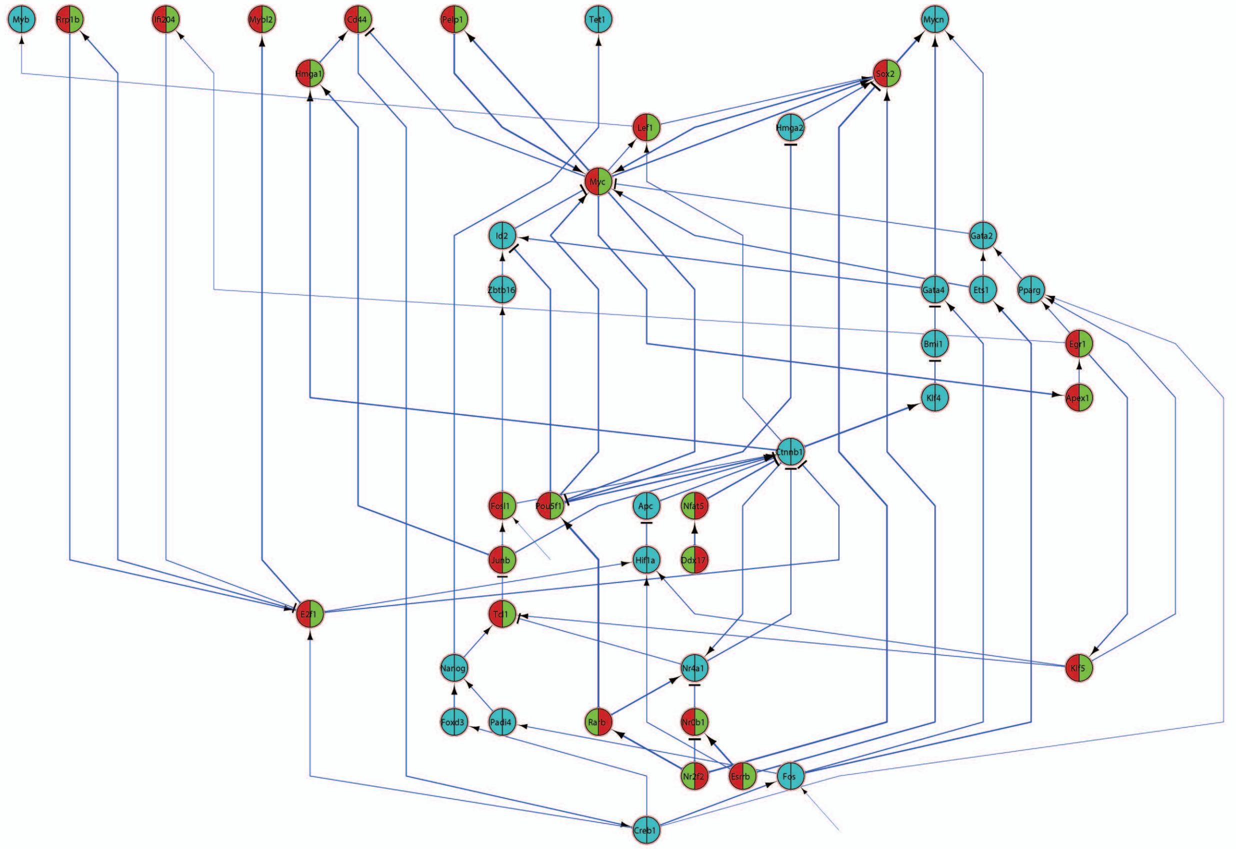

Supplementary Figure 5_2

e

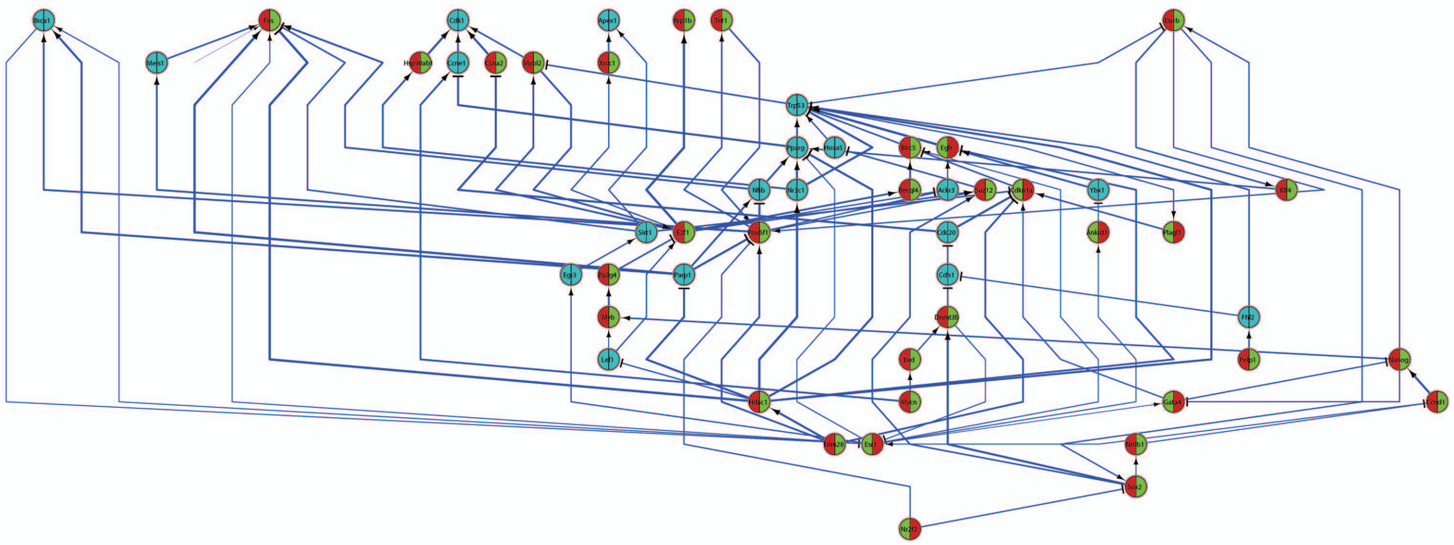

f

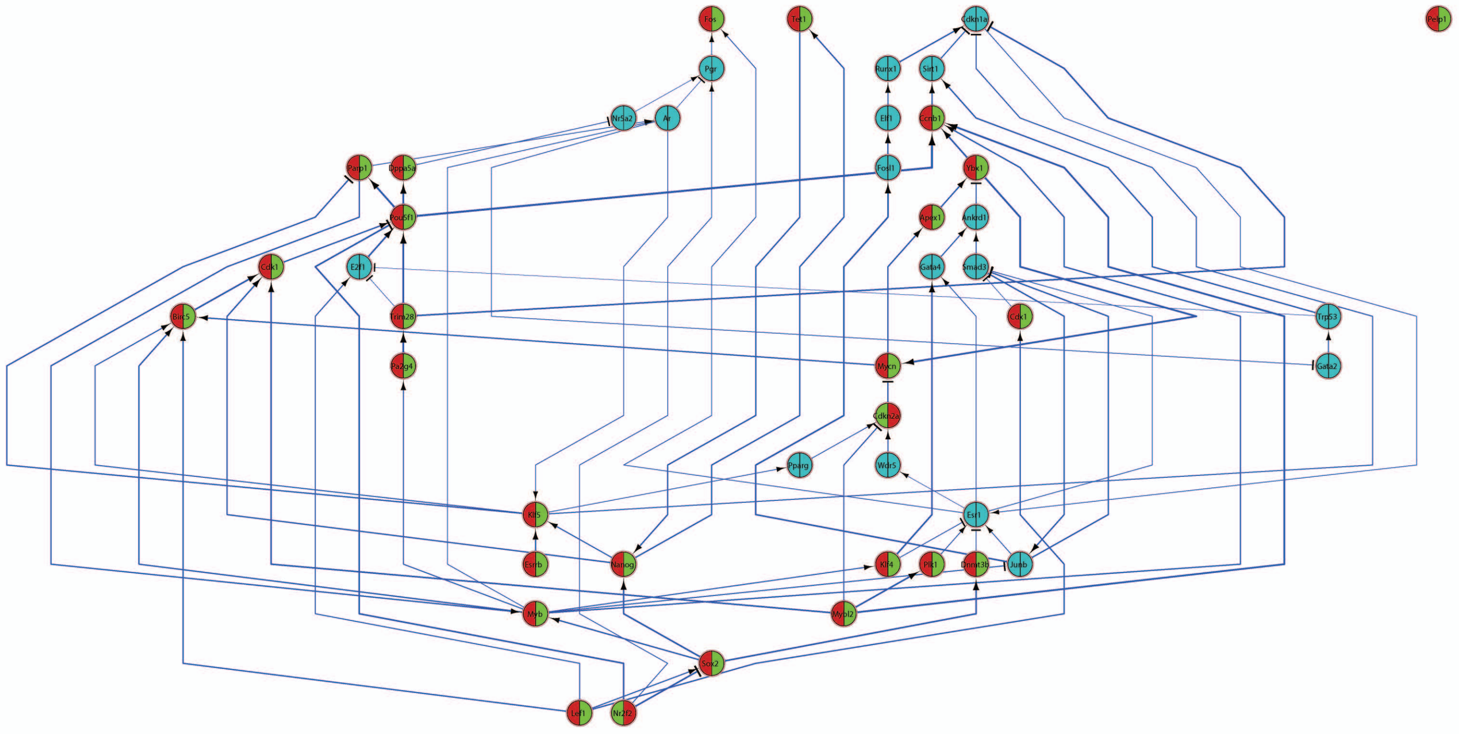

Supplementary Figure 5_3

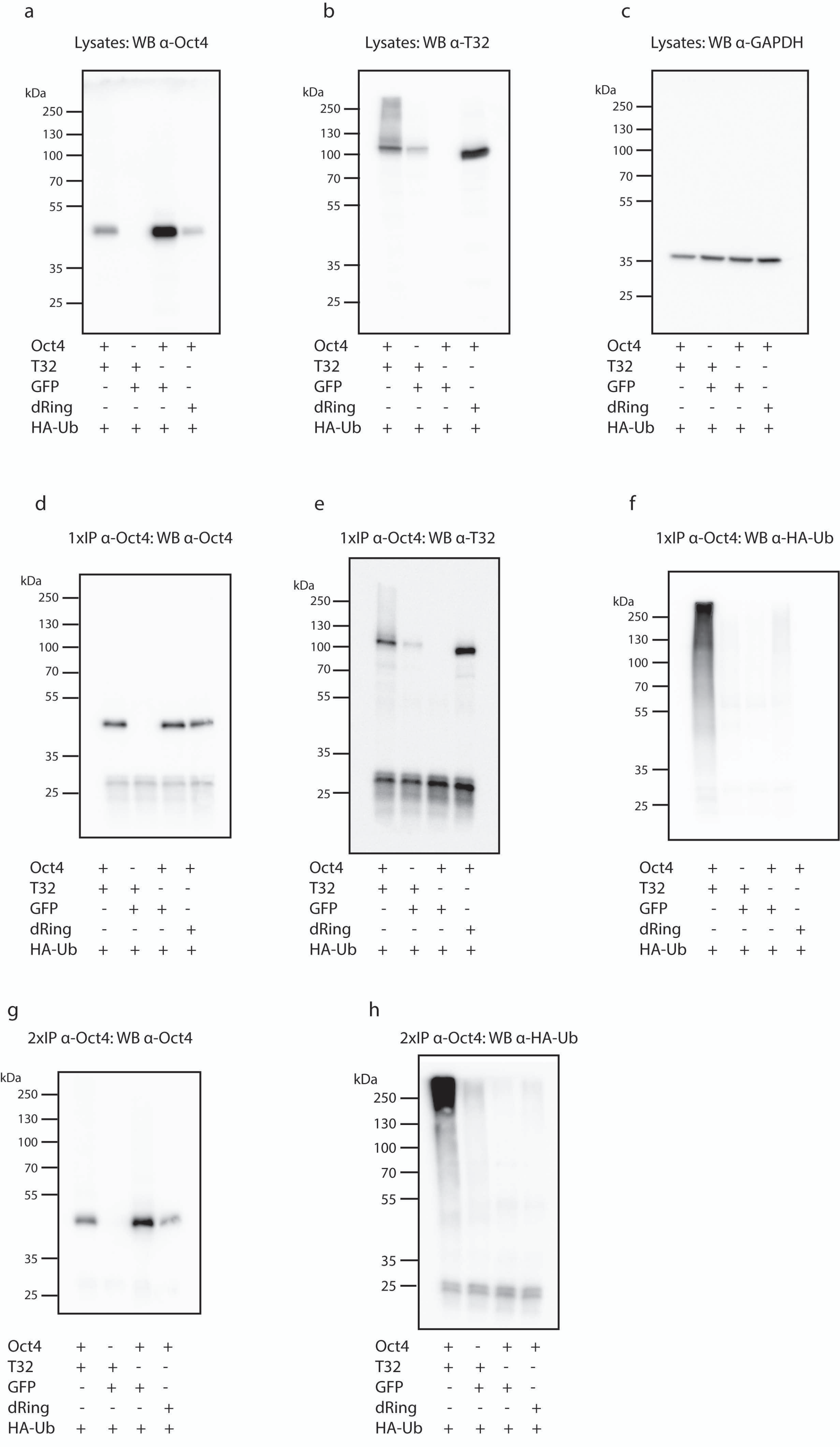

Supplementary Figure 6_1

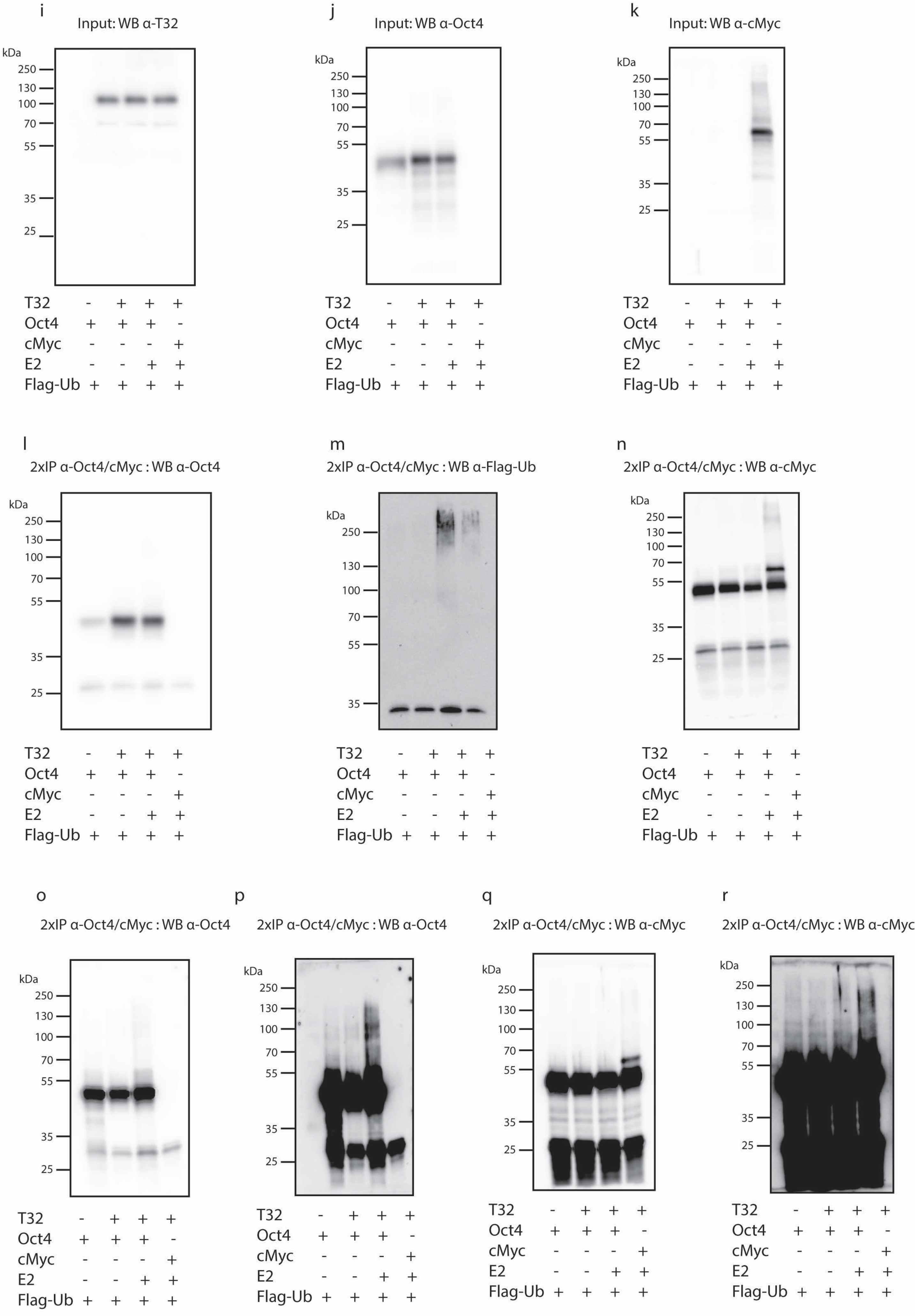

Supplementary Figure 6_2

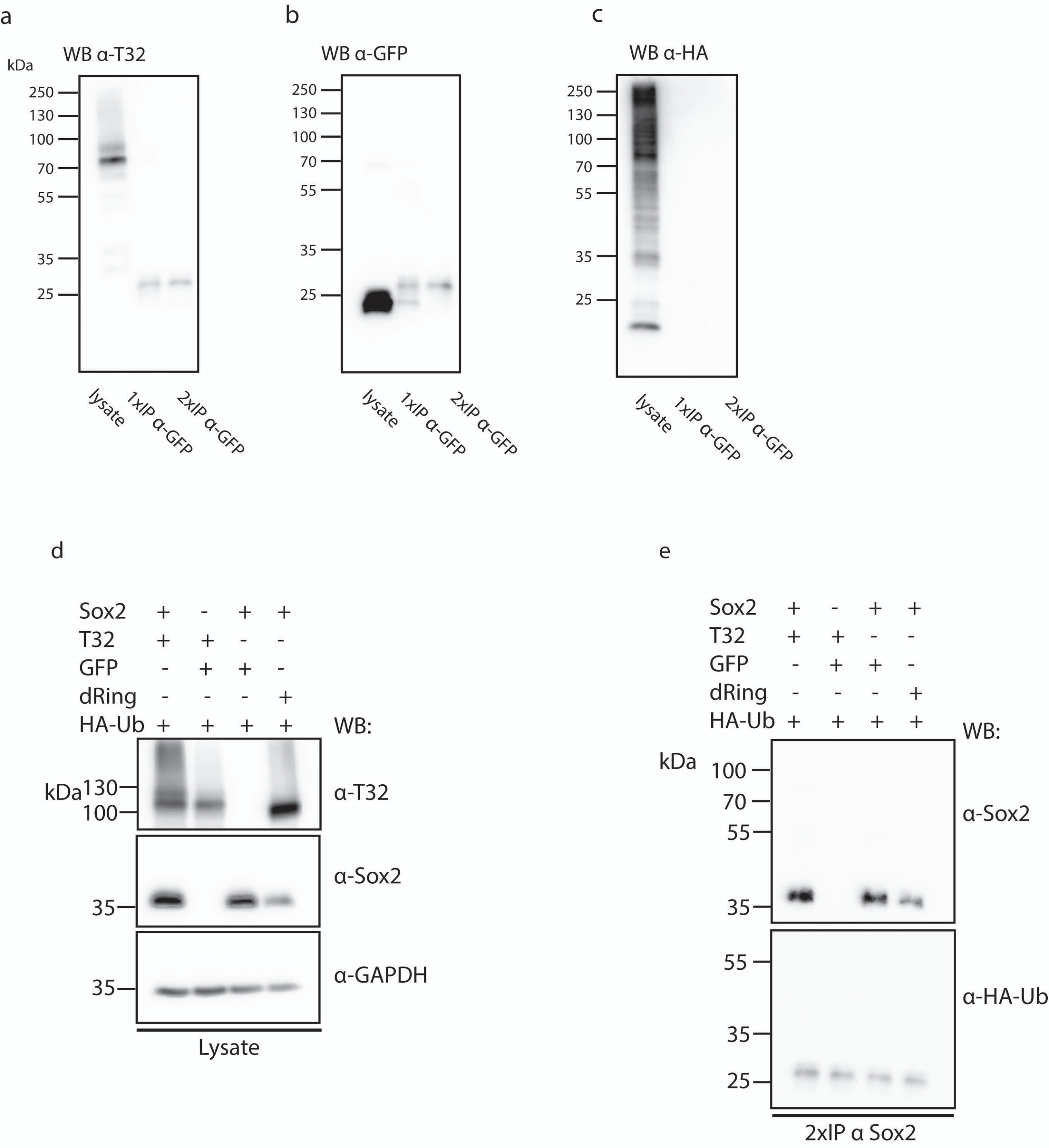

Supplementary Figure 7_1

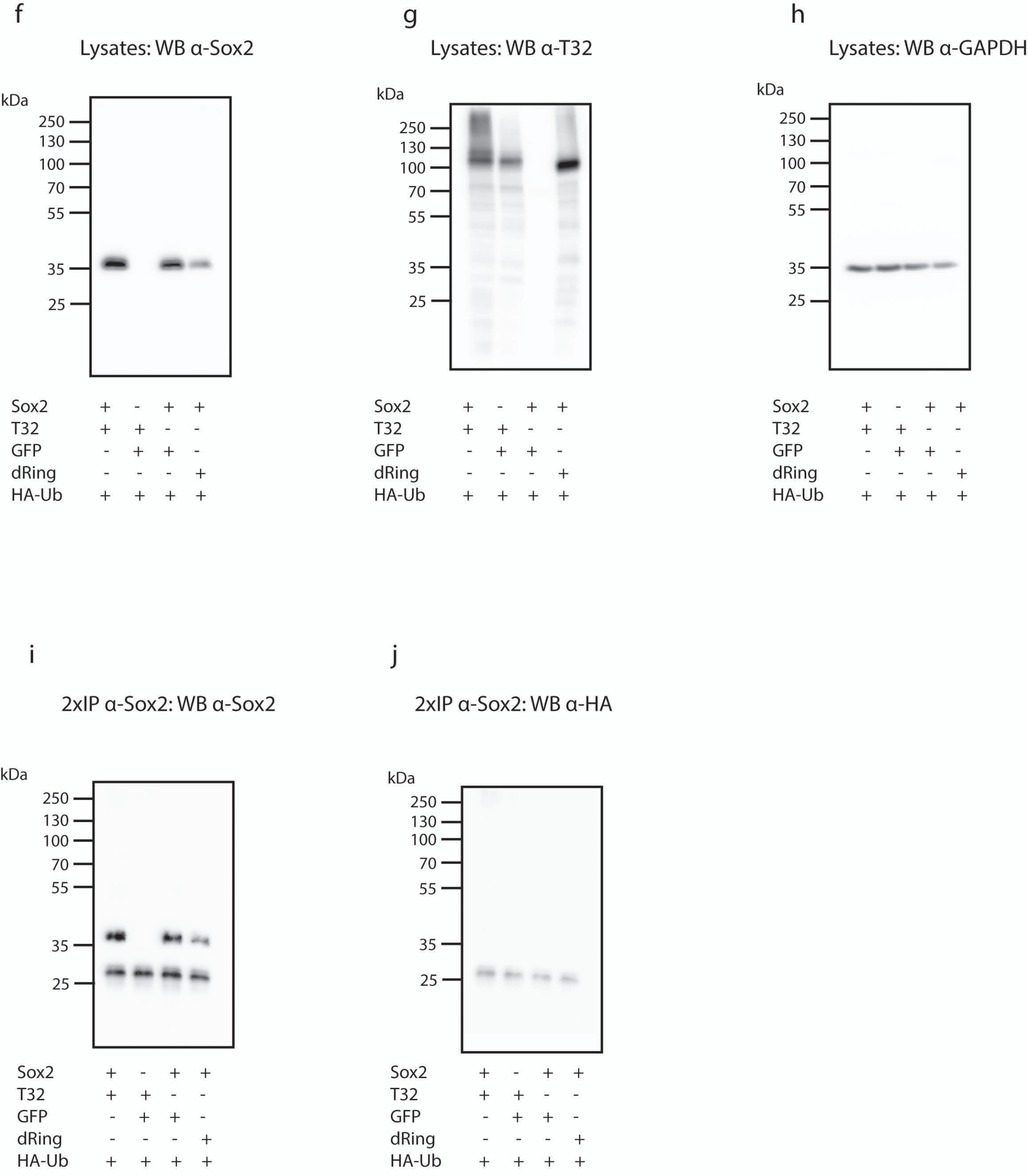

Supplementary Figure 7_2
